# Understanding the immune system architecture and transcriptome responses to southern rice black-streaked dwarf virus in *Sogatella furcifera*

**DOI:** 10.1038/srep36254

**Published:** 2016-11-02

**Authors:** Lin Wang, Nan Tang, Xinlei Gao, Dongyang Guo, Zhaoxia Chang, Yating Fu, Ibukun A. Akinyemi, Qingfa Wu

**Affiliations:** 1 School of Life Sciences, University of Science and Technology of China, Hefei, Anhui 230027, China; 2 Hefei National Laboratory for Physical Sciences at the Microscale, Bio-X Interdisciplinary Sciences, 443 Huang-Shan Road, Hefei, Anhui 230027, China

## Abstract

*Sogatella furcifera*, the white-backed planthopper (WBPH), has become one of the most destructive pests in rice production owing to its plant sap-sucking behavior and efficient transmission of Southern rice black-streaked dwarf virus (SRBSDV) in a circulative, propagative and persistent manner. The dynamic and complex SRBSDV-WBPH-rice plant interaction is still poorly understood. In this study, based on a homology-based genome-wide analysis, 348 immune-related genes belonging to 28 families were identified in WBPH. A transcriptome analysis of non-viruliferous (NVF) and viruliferous groups with high viral titers (HVT) and median viral titers (MVT) revealed that feeding on SRBSDV-infected rice plants has a significant impact on gene expression, regardless of viral titers in insects. We identified 278 up-regulated and 406 down-regulated genes shared among the NVF, MVT, and HVT groups and detected significant down-regulation of primary metabolism-related genes and oxidoreductase. In viruliferous WBPH with viral titer-specific transcriptome changes, 1,906 and 1,467 genes exhibited strict monotonically increasing and decreasing expression, respectively. The RNAi pathway was the major antiviral response to increasing viral titers among diverse immune responses. These results clarify the responses of immune genes and the transcriptome of WBPH to SRBSDV and improve our knowledge of the functional relationship between pathogen, vector, and host.

Many plant viruses that cause major world-wide agricultural problems are arthropod-borne and transmitted via insect vectors^1^. The white-backed planthopper (WBPH) *Sogatella furcifera* (Horvath) is an oligophytophagous insect that mainly feeds on rice plants. WBPH has emerged as a major rice pest in several Asian countries^2^. The insect pest damages rice plants by sucking the sap, resulting in reduced vigor, delayed tillering, stunted growth, yellow leaves, and shriveled grains; heavy infestation may also cause “hopper burn,” leading to the complete death of rice plants^3^. WBPH also serves as a vector for transmitting plant viruses, such as Southern rice black-streaked dwarf virus (SRBSDV). Similarly, two additional insects with sequenced genomes in the order Hemiptera, pea aphid (*Acyrthosiphon pisum*)^4^ and brown planthopper (*Nilaparvata lugens*, BPH)^5^, not only damage crops, but also serve as plant virus vectors^6^.

SRBSDV is a non-enveloped RNA virus of the genus *Fijivirus* with ten linear dsRNA genomic segments^7^; it is closely related to rice black-streaked dwarf virus (RBSDV). In recent years, SRBSDV has spread rapidly throughout southern China and northern Vietnam^8^. SRBSDV can be efficiently transmitted in a persistent-propagative manner by WBPH, but not by the rice pests brown planthopper (*Nilaparvata lugens*) and small brown planthopper (*Laodelphax striatellus,* SBPH)^6^, and it then enters the salivary glands through various routes for inoculation and transmission^9^. SRBSDV completes its life cycle in about 7 days^10^ and transmission to other locations is facilitated by the long-distance migration ability of WBPH^3^. The first line of defense against invading pathogens in insects is provided by physical barriers, such as the chitin exoskeleton and tracheal and intestinal epithelia^5^. After pathogens break the physical barrier, the immune system provides further protection in the form of humoral and cellular factors via the activation of immune signaling pathways, systemic production of effector molecules, and the activation of proteolytic cascades leading to coagulation and melanization. These innate immune pathways not only participate in antimicrobial activity, but have also been implicated in antiviral immunity, including Toll and IMD signaling pathways^11^,^12^, Janus kinase (JAK)–signal transducer and activator of transcription (STAT) signaling pathway^13^, autophagy^14^, and phenoloxidase cascade^15^,^16^. The small RNA interference (RNAi) pathway plays cellular antiviral roles by detecting virus-derived double-stranded RNA to direct the specific suppression of viruses^17^, while some viruses can also produce suppressors of RNAi^18^. Usually, persistent viral infection has limited adverse effects on its competent insect vector because the insect innate immune system is able to effectively modulate the persistent propagation of the virus in the body by preventing excessive viral accumulation^19^.

Assessing transcriptome changes as well as induced and suppressed transcripts in insect hosts in response to virus infection is an efficient strategy for studying the complicated interactions between viruses and insects, and is critical for inferring functional responses in insects. Although much progress has been made towards understanding the interactions between vectors and viral pathogens of human and animal diseases^20^,^21^, gene expression studies investigating plant virus–insect vector interactions remain limited, and only a few studies have characterized global transcriptome profiles of insect vectors fed on virus-infected plants^22^,^23^,^24^,^25^,^26^. The recently available genomes of WBPH allow us to identify immune genes and explore transcriptome changes in response to SRBSDV. In this study, the immune-related genes of WBPH were identified by homology-based searches and a relatively complete immune gene set was obtained. We also analyzed differentially expressed genes (DEGs) among individuals with various viral titers. We identified 1,906 genes with monotonically increasing expression (MIEGs) and 1,467 with monotonically decreasing expression (MDEGs) among viruliferous WBPH that exhibited viral titer-specific transcriptome changes. There were more immune system genes and cytoskeleton organization genes among MIEGs and MDEGs than DEGs in general, and the RNAi pathway was one of the major antiviral pathways. Taking together, this informative integrated immune analysis provides a deeper understanding of the innate immune system of WBPH, the biology of the virus within the insect host, and the host response to the virus.

## Results

### Homology-based identification of immune genes in WBPH

**Figure 1:**
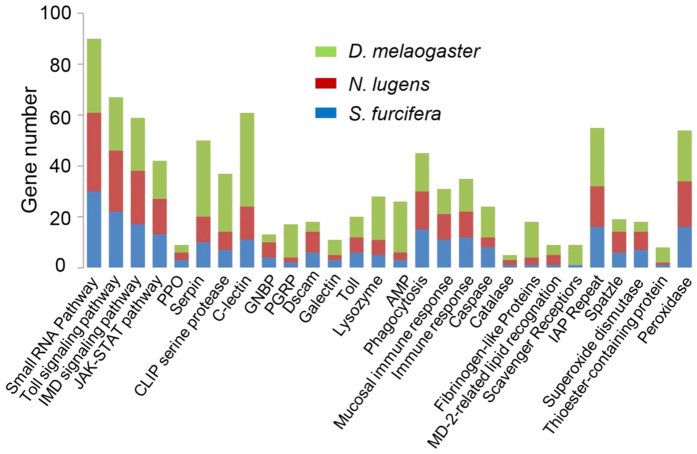
Comparison of immune genes families among three insects, *S. furcifera, N. lugens,* and *D. melanogaster*.

Innate immune responses have been well studied in holometabolous insect species, especially in dipterans (e.g., fruit flies and mosquitoes). By contrast, little is known about the immune responses in hemimetabolous insects. A total of 348 manually curated immune genes were identified in the WBPH genome based on a homology search of WBPH protein-coding genes against immune-related genes of model species (*Drosophila melanogaster* and *Anopheles gambiae*) and ImmunoDB (http://cegg.unige.ch/Insecta/immunodb). Among the 348 immune genes identified in WBPH, 231 were classified into 28 families and 4 functional groups (Fig. 1 and Fig. S1), which mainly included immune recognition families (PGRPs, GRPs, C-type lectins, SCRs, etc.), immune signaling pathways (Toll, IMD, JAK-STAT signaling pathways, RNAi pathway), immune response effectors (PPO, TEPs, AMPs, etc.), and others (SOD, catalases, peroxidases, etc.) (Tables S1 and S2), representing a relatively complete immune gene set for the species.

### Immune recognition families

Pathogen recognition is the first step in the defense against invading microorganisms, eliciting cellular and humoral responses. Pattern-recognition receptors (PRRs) bind conserved pathogen-associated molecular patterns (PAMPs) produced by pathogens^27^. Typical PRRs, including Peptidoglycan recognition proteins (PGRPs), β-Glucan binding proteins (GNBPs), lectins, hemolin, LPS-binding protein, gram-negative bacteria recognition protein, and thioester-containing protein (TEP), have been identified in insects. PGRPs are required to trigger signal transduction via the Toll and Imd pathways, leading to the activation of antimicrobial peptide (AMP) synthesis in insects. However, only two PGRPs (PGRP-LB and PGRP-LC) were identified in the WBPH genome, both of which were homologous to BPH PGRPs. C-type lectins, which recognize carbohydrates, play an important role in immune responses; for example, they activate the PPO cascade and recognize microorganisms to enhance microbial clearance^28^,^29^. In total, 13 C-type lectins were identified in the WBPH genome, including 2 C-lectins containing the mannose-binding motif E-P-N and 3 C-lectins containing the galactose-binding motif Q-P-D. Galectins are a family of lectins that contain an evolutionary conserved galectin-binding lectin specifically for galactoside sugar. Three galectins were identified in the WBPH genome (Table S1). TEPs are highly conserved proteins involved in the immune responses of animals; only one TEP was identified in WBPH and BPH. Thirteen scavenger receptors documented to contain multidomains that function as pattern recognition receptors in innate immunity were also identified, and these can be divided into three subfamilies, scavenger receptors A (SCRAs), scavenger receptors B (SCRBs), and scavenger receptors C (SCRCs), based on their functional domains (Fig. S2).

### Toll and Imd signaling pathways

The Toll pathway is primarily involved in defense against fungi and gram-positive bacteria. Spätzle (SPZ) is a cytokine activated in the Toll pathway that can bind to Toll receptor and further lead to AMP synthesis and the differentiation of certain hemocytes into lamellocytes^30^. The Imd pathway recognizes diaminopimelic acid-type peptidoglycan via the PGRP-LC receptor in pathogen invasion^31^. This receptor is membrane-bound and activates an intracellular pathway that turns on a set of immunity-related genes via Relish, overlapping with those induced by Dorsal and Dif. Orthologs of the key components of the Toll and IMD pathway were present in the WBPH genome (Table S1), including 5 SPZ genes, 7 toll receptors, 1 dorsal, 1 relish, and 1 Dif. All Toll receptors, except Toll-13, are composed of an extracellular LRR, transmembrane region, and intracellular TIR domain.

### RNA interference and JAK-STAT signaling pathways

The JAK-STAT pathway contributes to innate immunity by promoting the phagocytic activity of hemocytes as well as antiviral responses in insects^32^. One transcription activator (STAT)^33^, one cytokine receptor (Domeless), one Janus kinase (Hopscotch), one protein inhibitor of activated STAT (PIAS), and six suppressor of cytokine signaling (SOCS) genes of the JAK-STAT pathway were identified in the WBPH genome. RNAi destroys targeted RNAs via small RNA produced to inhibit gene expression. siRNA, which is central to RNAi, is triggered by double-stranded RNAs of viruses and these dsRNAs are recognized and cleaved by Dicer2 to generate siRNAs, which are then loaded into RNA-induced silencing complexes consisting of Argonaute-2 and other proteins^34^. The other small RNA antiviral response, the miRNA and piRNA pathway, targets viral nucleic acids and host gene regulation as well as transposon silencing and other epigenomic regulatory processes. In the WBPH genome, two Dicers (*Dicer1* and *Dicer2*), three Ago genes (*Ago1, Ago2*, and *Ago3*), *loqs, R2D2*, and *drosha* were identified, indicating that the RNAi pathway was highly conserved. Aubergine, which, like piwi, is expressed embryonically in the presumptive gonads, was also detected in WBPH.

### Melanization

We identified three prophenoloxidase (PPO) and seven CLIP genes each in WBPH, BPH, and ACYPI, which was fewer than the 23 CLIP genes in *D. melanogaster*, and 44 in *A. gambiae* (Fig. 1 and Fig. S1). In WBPH, four CLIP genes were homologous to proclotting enzymes and three were serine protease snake-like with a conserved N terminal CLIP domain. Serine protease inhibitors (serpins) present in insect hemolymph regulate the proPO activation cascade, functioning as negative regulators to avoid the excessive activation of the cascade^35^. We identified 10 serpins in WBPH, and all of them possessed a conserved serpin domain. Among the 10 serpins, 5 had putative signal peptides, implying that they were likely secreted; 5 serpins had serpin domains predicted to be non-cytoplasmic, and 2 serpins had serpin domains predicted to be cytoplasmic.

### Antibacterial peptide

AMPs in insects show lineage specificity both in copy numbers within a gene family and the presence/absence of an entire gene family^36^. The well-known attacin, cecropin, gloverin, lebocin, and moricin in lepidopteran insects and diptericin, drosocin, drosomycin, and metchnikowin in dipteran insects, are all absent in the hemipteran genome. Two copies of defensins were identified in WBPH, which has undergone extensive gene contraction. Several other effector genes, including reeler, lysozyme, and NOS, were present in the WBPH genome. Reeler is an immune-responsive gene that mediates the nodulation response upon bacterial infection. We identified two Reeler genes in the WBPH genome and five lysozymes in the WBPH genome, including one lysozyme showing the highest similarity to *N. lugens* c-type lysozymes and the other four lysozymes showing the highest similarities to *N. lugens* i-type lysozymes.

### **V**ariation in virus titers in WBPH fed on SRBSDV-infected rice plants

Vector competence is the inherent ability of a vector to acquire, circulate, replicate, and transmit a virus to a host^37^,^38^,^39^,^40^. Naturally, the SRBSDV virus could be detected in 25–83% of individuals in a WBPH population, referred to as virus carriers^6^,^8^,^41^, but the underlying mechanism determining viral titers *in vivo* is not well characterized. Because SRBSDV replicates within the insect, the immune system of WBPH plays important roles in determining the viral titer, which is positively associated with transmission efficiency and frequency.

**Figure 2:**
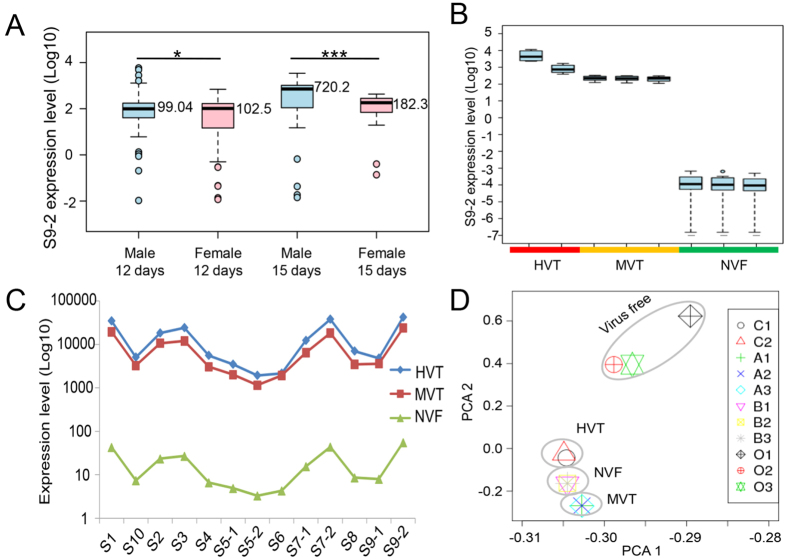
The expression levels of SRBSDV S9 segment in groups with different viral titers and a principal component analysis (PCA) of gene expression. (**A**) The expression level of SRBSDV S9 in male and female individuals on the 12^th^ and 15^th^ day, respectively. (**B**) The expression level of SRBSDV S9 for groups of males with three different viral titers on the 12^th^ day. (**C**) Viral gene expression patterns. The expression of all SRBSDV genes was graphed; lines correspond to each group. (**D**) Principal component analysis (PCA) across libraries based on gene expression. The first two components in the principal component analysis are shown, and the distance between the groups indicates the variance. *p < 0.05, ***p < 0.001.

On average, the RT-PCR band of the SRBSDV S9 segment could be observed in 67% and 69% of WBPH individuals collected on days 12 and 15, respectively, suggesting that insects that were fed on the same diseased plants could be divided into viruliferous and non-viruliferous individuals. The normalized abundance ratios of the SRBSDV S9 segment to RP49 transcripts was less than 0.01 in non-viruliferous individuals, as determined by real-time PCR. By contrast, the normalized abundance ratio of the SRBSDV S9 segment to RP49 transcripts was six orders of magnitude (0.01–5832) different among viruliferous individuals (Fig. 2A). The median SRBSDV genome copy number in viruliferous adults on day 15 was higher than that on day 12 (male: 720.21 *vs.* 99.04; female: 182.29 *vs.* 102.53), which is consistent with the assertion that WBPH is a persistent-propagative transmission vector for SRBSDV^42^. Interestingly, variation in viral titers was also observed between males and females. The viral titers in male individuals exhibited a broader distribution than that of females (Fig. 2A). In WBPH adults on the 12^th^ day, the amounts of the S9 segment were 0.206–5832 and 0.01–694 times higher than RP49 transcript levels in male and female individuals, respectively. Similarly, the relative amounts of virus in male and female adults on the 15^th^ day were 0.66–3444 and 0.137–430, respectively. The higher viral titers in male WBPH suggested that male WBPH was more efficient in transmitting SRBSDV compared with female WBPH of the same cohort (Student’s *t*-test, *p* = 0.045 and 1.79 × 10^−5^ on the 12^th^ and 15^th^ day, respectively). Female WBPH exhibit more complicated developmental and physiological processes, such as wing dimorphisms and fecundity, as well as a trade-off between the immune response and these processes^43^. To minimize interference by those physiological and development processes, only male WBPHs were used for the construction of RNA-seq libraries and data analysis.

### Altered transcriptome in WBPH fed on SRBSDV-infected rice plants

This quantitative relationship provides new insights into the biological parameters that may influence the spread of SRBSDV by WBPH. To investigate the DEGs among individuals that differ in viral titers, total RNAs were isolated from whole male insects. RNA-seq libraries representing groups with different viral titers (NVF: nonviruliferous, MVT: medial viral titer, HVT: high viral titer) with biological replicates from WBPH fed on SRBSDV-infected rice plants, and a control population that fed on healthy rice plants (Fig. 2B) were constructed for high-throughput sequencing. As expected, the abundance of viral SRBSDV sequences was 32.51% of total RNA-seq reads in the HVT group, on average (Table S3), and 21.39% in the MVT group, on average, and only 0.14% of viral SRBSDV sequences were identified in the NVF group (Table S3), consistent with the qualitative results of the qRT-PCR analysis (Fig. 2C).

**Figure 3:**
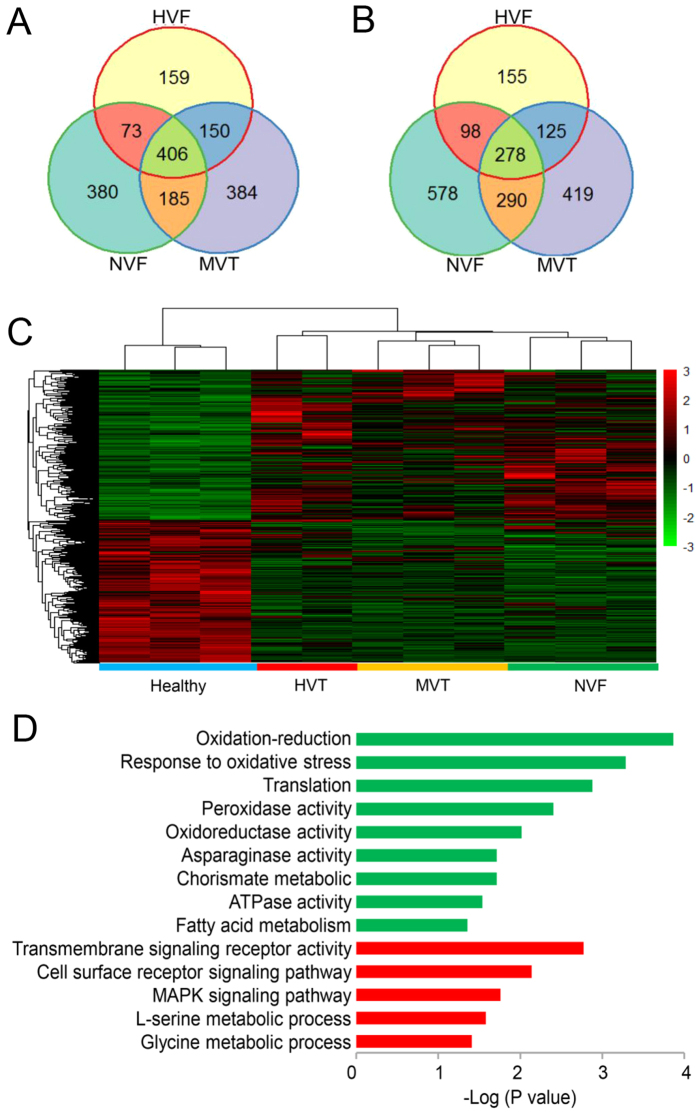
Differentially expressed genes (DEGs) and a GO enrichment analysis. (**A**) Venn diagram showing the number of genes commonly and differentially expressed in the HVT, MVT, and NVF groups for upregulated (**A**) and downregulated genes. (**B**,**C**) Global expression patterns of 3,676 DEGs using a hierarchical clustering analysis. (**D**) GO enrichment analysis of co-regulated DEGs. The different colors represent different DEGs (down-regulated genes are marked green and up-regulated genes are marked red).

To obtain an overview of the relationships among the 11 samples, a principal component analysis (PCA) was performed using the total gene expression profiles. As expected, we found that SRBSDV-infected groups and the SRBSDV-free group showed high variance, while the biological replicates within each group showed low variance (Fig. 2D). Then, we used edgeR to detect DEGs with a cutoff fold change of ≥2 and FDR <0.05. Compared with the healthy control group, we identified 1,444 (656 up-regulated genes and 788 down-regulated genes), 2,237 (1,112 up-regulated genes and 1,125 down-regulated genes) and 2,288 DEGs (1,244 up-regulated genes and 1,044 down-regulated genes) in the HVT group, MVT group, and NVF group (Fig. 3A,B), respectively. In order to investigate the comprehensive expression landscape of altered gene regulation during SRBSDV infection, a hierarchical clustering analysis was performed for all of the 3,676 DEGs detected in the three comparisons (Fig. 3C). The gene expression profiles of sample replicates for each group were obviously grouped in distinct clusters. The HVT, MVT, and NVF groups exhibited relatively similar expression patterns, differing markedly from that of the healthy control group (Fig. 3C). These patterns might reflect the direct effect of virus infection or indirect effect of feeding on the SRBSDV-infected rice plants.

In addition, 684 DEGs, including 278 up-regulated genes and 406 down-regulated genes, were common to all three groups with differences in viral titers, suggesting the extent of regulated genes was dramatic in WBPH that fed on SRBSDV-infected rice plants, regardless of the viral titers in insects. Gene ontology (GO) and KEGG enrichment analyses were conducted for all common DEGs using a hypergeometric test. Up-regulated genes were significantly (*p* < 0.05) enriched in amino acid metabolism and receptor signaling pathway functions, including glycine and l-serine metabolic processes, transmembrane signaling receptor activity, the cell surface receptor signaling pathway, and the MAPK signaling pathway (Fig. 3D). The MAPK signaling pathway, which mediates the nuclear response of cells to changes in the environment, serves as a central pathway involved in the response to HBV (hepatitis B virus) infection^44^. Among down-regulated genes, those involved in primary metabolism (fatty acid metabolism, asparaginase activity, translation, chorismate metabolic, and ATPase activity) and oxidoreductase activity (oxidation-reduction process, response to oxidative stress, and peroxidase activity) were overrepresented (Fig. 3D). These results were consistent with those of previous studies on the effects of virus infection indicating that SRBSDV induces the down-regulation of primary metabolism-related genes in WBPH^24^. The down-regulation of lipid metabolism was also observed in whiteflies infected by Tomato yellow leaf curl China virus^23^. Effects on lipid metabolism have also been reported in other virus infections, e.g., human cytomegalo virus infection results in increases in the flux of the fatty acid biosynthesis pathway genes essential for optimal viral growth in fibroblasts. In addition, genes involved in the oxidation-reduction process, the rate-determining factor for virus inactivation^45^, were also down-regulated in virus-infected male adults, which provides new insight into the interaction between SRBSDV and the competent vector WBPH.

### Suppression of cellular and humoral immune responses in WBPH fed on SRBSDV-infected rice plants

Among the DEGs in the SRBSDV-infected groups, 27 immune-related DEGs were identified in the HVT, MVT, and NVF groups (Table S4). First, 5 of 17 peroxidases and 1 of 7 superoxide dismutases (SODs) were significantly down-regulated. Peroxidases and SODs play a major role in the defense against reactive oxygen species (ROS) in insect innate immune responses^46^. Recently, peroxidases and SODs have been reported to be differentially expressed in *L. striatellus* infected with RSV^47^. Thus, these results implied that peroxidases and SODs were likely to participate in the immune responses of WBPH against viruses. The Duox enzyme responsible for the mucosal immune response was down-regulated, suggesting that ROS immunity was suppressed in the midgut and salivary gland. Defensins, belonging to a diverse group of antimicrobial peptides with pronounced antimicrobial activity, were also down-regulated in the infected groups. Next, a gene encoding Spätzle, the ligand of Toll signaling, was down-regulated, indicating that Toll signaling may be inhibited in high-level SRBSDV-infected males compared to control individuals. In addition, we discovered several DEGs involved in the melanization defense response, including two down-regulated CLIP-domain serine proteases and three up-regulated serpins. CLIPs can activate the melanization defense response by producing phenoloxidases, while serpins act as inhibitors of the pathway to prevent insects from excessive melanization. The phenoloxidase cascade is implicated in the control of virus infections in SFV-infected mosquitos, and SFV can express a PO cascade inhibitor to block PO activity and thus enhance virus spread^15^,^16^. Therefore, the melanization defense response was inhibited during SRBSDV infection and contributes to spread of SRBSDV in WBPH.

### Virus titer-dependent differences in transcript expression profiles

**Figure 4:**
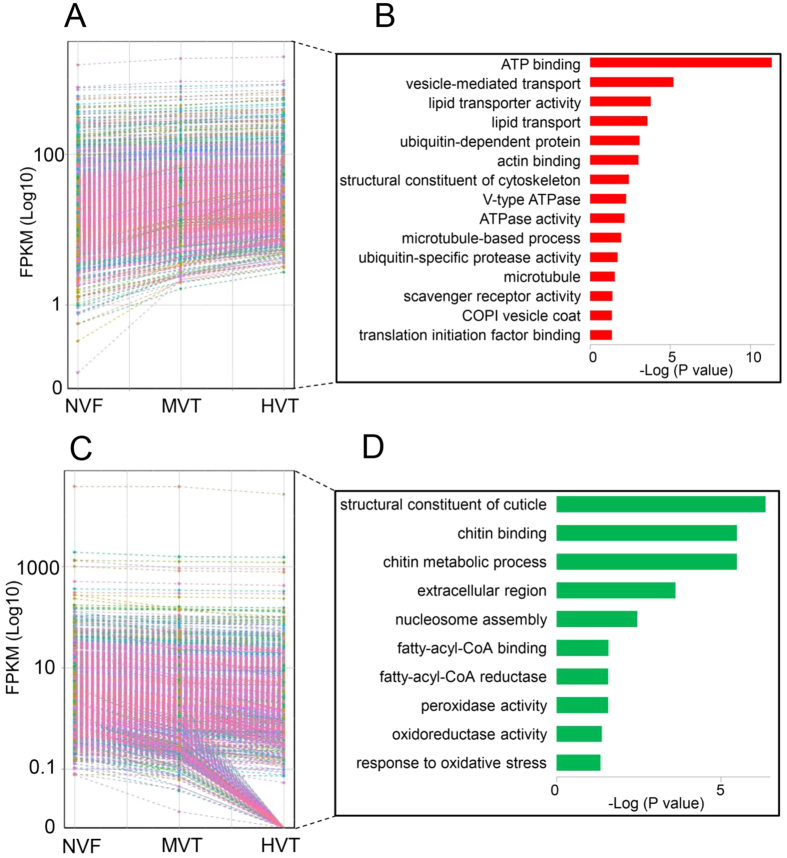
Gene expression patterns of MIEGs and MDEGs and a GO enrichment analysis. (**A**) The expression of each gene was plotted against the viral titer from NVF to HVT. (**B**) GO enrichment analysis of MIEGs. (**C**) The expression of each gene was plotted against the viral titer from NVF to HVT. (**D**) GO enrichment analysis of MDEGs.

Compared with the SRBSDV-free group, the suppression of cellular and humoral immune responses was observed in WBPH fed on SRBSDV-infected rice plants. However, variation in transcript expression profiles was also discovered among the HVT, MVT, and NVF groups, and these differences might be relevant to the SRBSDV-WBPH interaction. In order to identify the monotonically differentially expressed genes that might contribute to differences in viral titers among WBPH fed on SRBSDV-infected rice plants, we selected MIEGs and MDEGs in the NVF, MVT, and HVT groups, from low to high SRBSDV viral titers. In total, 1,467 MDEGs and 1,906 MIEGs were identified (Fig. 4A,C). MDEGs were enriched for functions in chitin metabolism (chitin metabolic process, chitin binding, structural constituent of cuticle) and oxidation-reduction processes (e.g., response to oxidative stress and oxidoreductase activity) (Fig. 4B), MIEGs were enriched for cytoskeleton organization (microtubule-based process, microtubule, structural constituent of cytoskeleton and actin binding), lipid metabolism, and ubiquitin-specific protease activity (Fig. 4D).

Invasion and dissemination of the virus are considered the infection barrier and spread-limiting steps in the body of vector insects^48^,^49^. The first line of defense is provided by physical barriers, such as the chitin exoskeleton and tracheal and intestinal epithelia that protect the insect against entry by invading pathogens^50^,^51^. The down-regulated genes in the MDEG group were enriched for functions in the chitin metabolic process, chitin binding, and structural constituent of cuticle (Fig. 4D), which might facilitate SRBSDV invasion. Cytoskeleton-dependent intracellular transport is a common strategy for viral transport to intracellular destinations, and microtubules are required for intracellular mature virus assembly and release^49^. Association with microtubules is necessary for the release of Rice gall dwarf virus (RGDV) from cultured cells^49^. The tubular structures constituted by Pns10 of Rice dwarf virus (RDV) facilitate the spread of viruses in the vector *N. cincticeps*^52^. SRBSDV, RGDV, and RDV belong to the *Reoviridae* family, and it is possible that MIEGs involved in cytoskeleton organization play a role in SRBSDV dissemination via the cytoskeleton regulatory pathway in WBPH.

### RNAi pathway was up-regulated in response to SRBSDV replication

The general cellular and humoral immune responses were suppressed in WBPH fed on SRBSDV-infected rice plants; 66 immune genes were up-regulated in the NVF, MVT, and HVT groups and identified as virus MIEGs. These genes were distributed in various immune pathways, including the RNAi pathway (15 genes), the Toll pathway (2 genes), the IMD pathway (4 genes), melanization (6 genes), pattern recognition receptors (5 genes), antimicrobial humoral response (11 genes), mucosal immune response (3 genes), and caspase (2 genes). This demonstrated that diverse immune responses were triggered and these responses increased by SRBSDV infection and accumulation *in vivo*.

**Figure 5:**
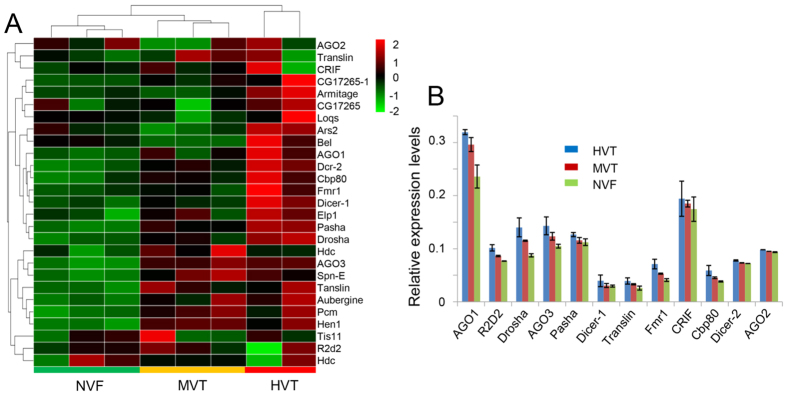
Global expression patterns of 28 immune genes in the RNAi pathway and an RT-qPCR assay of immune genes. (**A**) Global expression patterns of 28 immune genes in the RNAi pathway using a hierarchical clustering analysis. (**B**) Quantitative RT-PCR analyses of the expression of RNAi mRNAs. The levels were normalized using RP49 transcripts as an internal control. The mean RT-qPCR assay results ± the standard deviations of three biological replicates are shown.

The RNAi pathway is the classical antiviral immune pathway and is able to effectively modulate the replication of persistent propagative transmitted viruses^37^. Among all 27 genes of the RNAi pathway identified in WBPH, 15 were identified as MIEGs showing enhanced expression as the viral titer increased, among the NVF, MVT, and HVT groups (Fig. 5A), indicating that the RNAi pathway was significantly up-regulated (Hypergeometric test, *p* = 1.13 × 10^−9^). The 15 genes, including *dcr-1, dcr-2, ago1,* and *ago3*, are important components in the siRNA, miRNA, and piRNA pathways. The expression profiles of all 27 RNAi pathway genes were also investigated, and a hierarchical clustering analysis based on the expression level of these genes showed that biological replicates in each group formed obvious clusters and the distance between the NVF group and HVT group was the largest (Fig. 5A), suggesting the global expression pattern of all RNAi pathway genes was consistently up-regulated by SRBSDV replication and accumulation. Twelve genes in the RNAi pathway were randomly selected and the expression levels of these genes were validated by qRT-PCR (Fig. 5B). These results showed that the RNAi pathway serves as a major antiviral response and this response increases gradually with the accumulation of SRBSDV in WBPH.

Activation of the siRNA pathway is characterized by the production of viral siRNAs derived from the viral genome^50^. Deep sequencing of small RNAs showed that the normalized number of viral siRNAs was 16 and 50,207 reads per million (RPM) in the NVF and MVT groups, respectively, and was positively correlated with the amount of virus genome copies detected by RNA-seq and qRT-PCR. The typical sizes of viral siRNAs were 21 nt (43.3–50.6%) and 22 nt (29.3–32.8%) (Fig. S3), consistent with previous findings in cultured cells^51^. The (+) and (−) viral siRNAs were almost equal and distributed evenly across all 10 segments (Figs S4 and S5). Taking together, these results demonstrated that the RNAi pathway was up-regulated by SRBSDV replication and accumulation and all 10 SRBSDV dsRNA genome segments are equally accessible to the dicing machinery of the RNAi pathway during viral replication.

## Discussion

Many viral pathogens that cause significant global health and agricultural problems are arthropod-borne and transmitted via an insect vector^53^. These viruses are maintained in nature through alternating virus replication in susceptible hosts and insect vectors. WBPH serves as a vector for transmitting SRBSDV; however, the interaction between SRBSDV and WBPH is still poorly understood. Understanding the architecture of the immune system of WBPH is critical to study the molecular mechanisms of viral transmission by insect vectors. In this study, genome-wide identification of WBPH immune genes was performed based on the assembled WBPH genome and transcriptome data. In total, 348 immune genes were identified in WBPH using a homology-based approach. The number of immune genes in WBPH was 88.0% and 89.1% of the numbers observed in fruit flies and mosquitoes, respectively. A comparison of the immune repertoire among insects revealed that WBPH clustered with BPH, suggesting that both insects have a depauperate immune repertoire. Social insects also exhibit fewer immune genes and clustered with hemipteran insects. They instead typically exhibit group-based defenses (‘social immunity’) that should reduce selective pressures on immune genes^54^. Fewer immune genes observed in WBPH and BPH genomes may indicate that this deficiency is a more general characteristic of Delphacidae insects. WBPH and BPH possess a special phloem-sap sucking style to avoid pathogens, which might reduce selective pressures on immune genes.

The complete and well-annotated genome of WBPH provides valuable information for transcriptome analyses to dissect the complicated interactions between SRBSDV and its vector WBPH. The comparative gene expression analysis in WBPH revealed that feeding on SRBSDV-infected rice plants has a significant impact on gene expression. A functional analysis of these DEGs revealed that primary metabolism-related genes and oxidoreductase activity genes were significantly down-regulated (Fig. 3D), consistent with previous studies^24^. By contrast, genes involved in the cellular and humoral immune responses in WBPH fed on SRBSDV-infected rice plants were suppressed, especially genes related to ROS immunity and melanization defense. The pattern of immune responses observed in this study can be attributed to the unique methods employed for the analysis. First, the RNA-seq libraries were prepared from only male WBPH as compared to mixed populations used in previous studies^24^. Analyses based on a single sample population are more specific and represent the expression of the genes in response to viral infection; in contrast, females have more complicated developmental and physiological processes, such as wing dimorphisms, fecundity, and trade-offs between these traits and immune responses. Second, the WBPH samples were classified into NVF, MVT, and HVT groups with distinct viral titers to determine the effects of viral titers on the transmission and propagation efficiency of the insect vector.

To gain insights into the viral titer-specific transcriptome changes in WBPH, we identified 1,906 MIEGs and 1,467 MDEGs exhibiting strict increases and decreases in expression, respectively, with respect to viral titers among WBPH fed on SRBSDV-infected rice plants. These WBPH were treated under identical conditions, suggesting that MDEGs and MIEGs are mainly related to changes in viral replication and accumulation, and not by indirect effects of diseased rice plants. Most MDEGs and MIEGs were missed in previous DEG analyses because the fold change values of these genes were less than two. It is notable that genes involved in chitin metabolism were enriched in the set of MDEGs, while genes involved in cytoskeleton organization were enriched in the set of MIEGs, suggesting that SRBSDV infection consistently reorganizes the structure of intestinal epithelia and intracellular cytoskeleton-dependent intracellular transport in WBPH to facilitate SRBSDV invasion and dissemination. Most interestingly, 66 immune genes were identified as MIEGs, indicating these immune pathways accumulated in response to SRBSDV replication. The RNAi pathway is the typical response; 56% (15/27) of genes in the pathway were identified as MIEGs, which is consistent with the previous finding that the RNAi pathway plays a crucial role in the modulation of the persistent propagation of SRBSDV in its competent vectors^48^. Considering that many cellular and humoral immune response genes were suppressed in the DEG analysis, the immune interaction between WBPH and SRBSDV is very complicated. Investigations of tissue- or organ-specific immune transcriptomes during SRBSDV infection are necessary to understand the biology of the virus within the insect host and the host response to the virus.

## Methods

### Sample preparation and RNA isolation

The second-instar WBPH nymphs were fed on SRBSDV-infected rice plants. After 2 days, all nymphs were transferred and maintained on healthy rice seedlings in a man-made climate chamber with a temperature of 26 °C, 70% relative humidity, and a photoperiod of 16 hours of light and 8 hours of dark. The control group was reared on healthy rice seedlings consistently under the same conditions. WBPH were collected on the 12^th^ or 15^th^ day, respectively. Total RNA was extracted using TRIzol according to the manufacturer’s instructions individually. The SRBSDV genome copy numbers in WBPH individuals were determined on the 12^th^ and 15^th^ day after the first exposure to diseased plants.

### RT-PCR and real-time reverse-transcription PCR

RNA was treated using Baseline-ZERO™ DNase (Epicentre, Madison, WI, USA) to remove genomic DNA contamination before use for cDNA synthesis. Total RNA (~100 ng) was reverse-transcribed into cDNA using the RevertAid First Strand cDNA Synthesis Kit (Fermentas, Waltham, MA, USA) and random primers (Promega, Madison, WI, USA) following the manufacturer’s instructions. Polymerase chain reaction (PCR) was used to detect S5 or S10 of SRBSDV and the products were run on an agarose gel (S5-F ttacaactggagaagcattaacacg, S5-R atgaggtattgcgtaactgagcc; S10-F cgcgtcatctcaaactacag, S10-R tttgtcagcatctaaagcgc. Quantitative PCR (qPCR) was performed using the LightCycler 96 (Roche) with FastStart Essential DNA Green Master Mix, and the *RP49* gene of WBPH was used as an internal control to measure the expression level of S9-2 of SRBSDV (S9-2-FAATCCTTGCTGTATATCATTCTT, S9-2-R TACCTCCATTGAACACTTGT; SF-RP49-F CTGGCGTAAACCAAAGGGTA, SF-RP49-R TCTGCATCATCAGCACTTCC). All reactions were performed in triplicate, and the expression level of S9-2 was calculated using the 2^−ΔΔCt^ method^55^.

### RNA-seq library construction and sequencing

The 12-day infected male and female WBPH were divided into 21 groups according to the expression level of S9-2 (Fig. 2A,B). About 10 μg of total RNA from each sample was treated with Baseline-ZERO™ DNase (Epicentre). RiboMinus RNA was isolated using the Ribo-Zero™ Magnetic Gold Kit (Human/Mouse/Rat) (Epicentre) using 4 additional probes targeting the ribosomal RNA of WBPH (5′-biotin-AAGCGACGTCGCTATGAACGCTTGGCCGCCACAAG-3′, 5′-biotin-ATCCATTCATGCGCGTCACTAATTAGATGACGAGG-3′, 5′-biotin-CTTCGGCAGGTGAGTTGTTACACACTCCTTAGCGG-3′, 5′-biotin-TACCGCGGCTGCTGGCACCAGACTTGCCCTCCAAT-3′) according to the kit instructions, and the libraries were then constructed according to previously described methods^56^ and sequenced using the HiSeq X10 platform (Illumina, San Diego, CA, USA).

### Identification and classification of immune genes in ***S. furcifera***

The sequences of immune genes in *D. melanogaster* and other insect immune genes were downloaded from the immunology database at http://cegg.unige.ch/Insecta/immunodb or obtained from previous studies, and were downloaded as queries. These immune-related sequences were used to search for immune genes in WBPH. A local tblastn search with an E-value cut-off of 10^−5^ was performed to collect putative immune genes. Finally, the edited sequences were manually confirmed against the NCBI curated and conserved domains (CDD) database using BLASTx. Domain architecture was analyzed using the SMART (http://smart.embl-heidelberg.de/), NCBI CDD database (http://www.ncbi.nlm.nih.gov/Structure/cdd/docs/cdd_search.html) and PROSITE (http://au.expasy.org/prosite/). Transmembrane domains were analyzed using TMHMM server v. 2.0 (http://www.cbs.dtu.dk/services/TMHMM/). Signal peptides were analyzed using SignalP3.0 (http://www.cbs.dtu.dk/services/SignalP/).

### RNA sequencing and differential expression analysis

In this study, WBPH transcriptome data from male adults infected with high and low levels of SRBSDV were used. Low-quality bases in the RNA sequencing data were trimmed using a PERL script, and adaptors were removed using Cutadapt (version 1.3). The remaining reads were mapped against the reference sequences of rRNA and SRBSDV using Bowtie2 with default parameters, and reads matching rRNA or SRBSDV were discarded. The filtered reads were mapped to the assembled WBPH genome using STAR^57^ with the parameters --runThreadN 8 –outFilterMultimapNmax 20 –outFilterMismatchNmax 4 –outFilterIntronMotifs RemoveNoncanonical. Cuffdiff2 was used to calculate FPKM (fragments per kilobase of exon per million fragments mapped) to evaluate the gene expression levels. In the analysis of the Cuffdiff2 results, *p* < 0.05 and |log2(fold change)| >1 were set as the thresholds to identify differentially expressed genes (DEGs). Those genes exhibiting strict monotonically increasing or decreasing expression as viral titers increase among the NVF, MVT, and HVT groups were characterized as candidate MIEGs or MDEGs. These candidates were further filtered according to whether |FPKM_NVF_-FPKM_MVT_| or |FPKM_MVT_-FPKM_HVT_| <0.5. The KEGG pathway mapping tool was used to map DEGs to KEGG pathways. GO Term Mapper was used to generate the GO slim annotation of *D. melanogaster* to retrieve immune genes. Expression-based sample clustering was performed using the heatmap function implemented in the R package gplots.

### Accession numbers

The transcriptome (RNA-Seq) data described in this study were deposited at SRA database of NCBI under accession number SRR4379960, SRR4379969-73, SRR4379975-78 and SRR4379996.

## Additional Information

**How to cite this article**: Wang, L. *et al*. Understanding the immune system architecture and transcriptome responses to southern rice black-streaked dwarf virus in *Sogatella furcifera. Sci. Rep.* **6**, 36254; doi: 10.1038/srep36254 (2016).

**Publisher’s note:** Springer Nature remains neutral with regard to jurisdictional claims in published maps and institutional affiliations.

## Supplementary Material

Supplementary Information
