# Supplementary material for: Understanding the immune system architecture and transcriptome responses to southern rice black-streaked dwarf virus in Sogatella furcifera
Source: Sci Rep. 2016 Nov 2;6:36254. doi: 10.1038/srep36254 (PMC5090245; doi:10.1038/srep36254)
Supplement: Supplementary Information [file srep36254-s1.pdf]

Understanding the immune system architecture and transcriptome responses to southern rice black-streaked dwarf virus in *Sogatella furcifera*

Lin Wang[1,*], Nan Tang[1,*], Xinlei Gao[1], Dongyang Guo[1], Zhaoxia Chang[1], Yating Fu[1], Ibukun A. Akinyemi[1], Qingfa Wu[1, 2, &]

1. School of Life Sciences, University of Science and Technology of China, Hefei, Anhui 230027, China
2. Hefei National Laboratory for Physical Sciences at the Microscale, Bio-X Interdisciplinary Sciences, 443 Huang-Shan Road, Hefei, Anhui 230027, China

*Co-first author
[&] Correspondence: wuqf@ustc.edu.cn

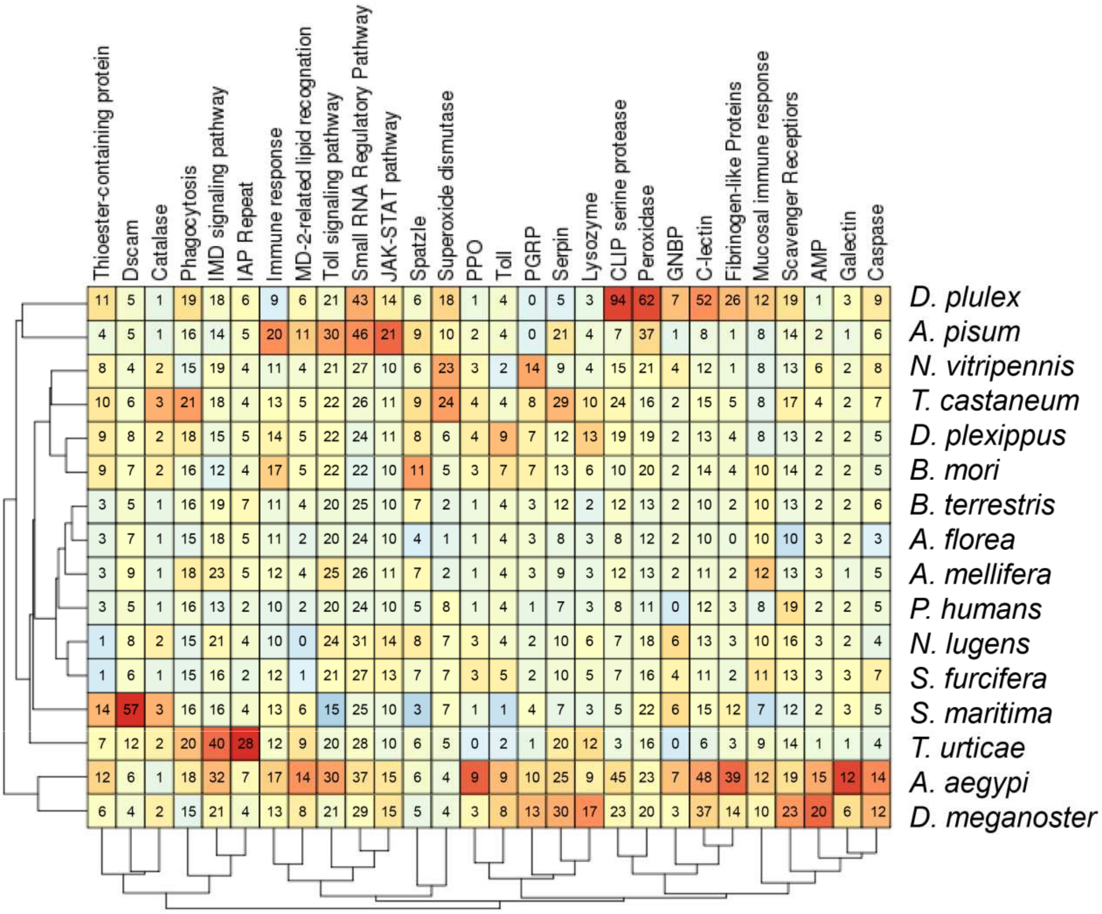

Figure S1. Heatmap of immune genes belonging to 28 families among 16 insects and arthropod. The clustering analysis was performed using Euclidean distances based on the number of genes within these families.

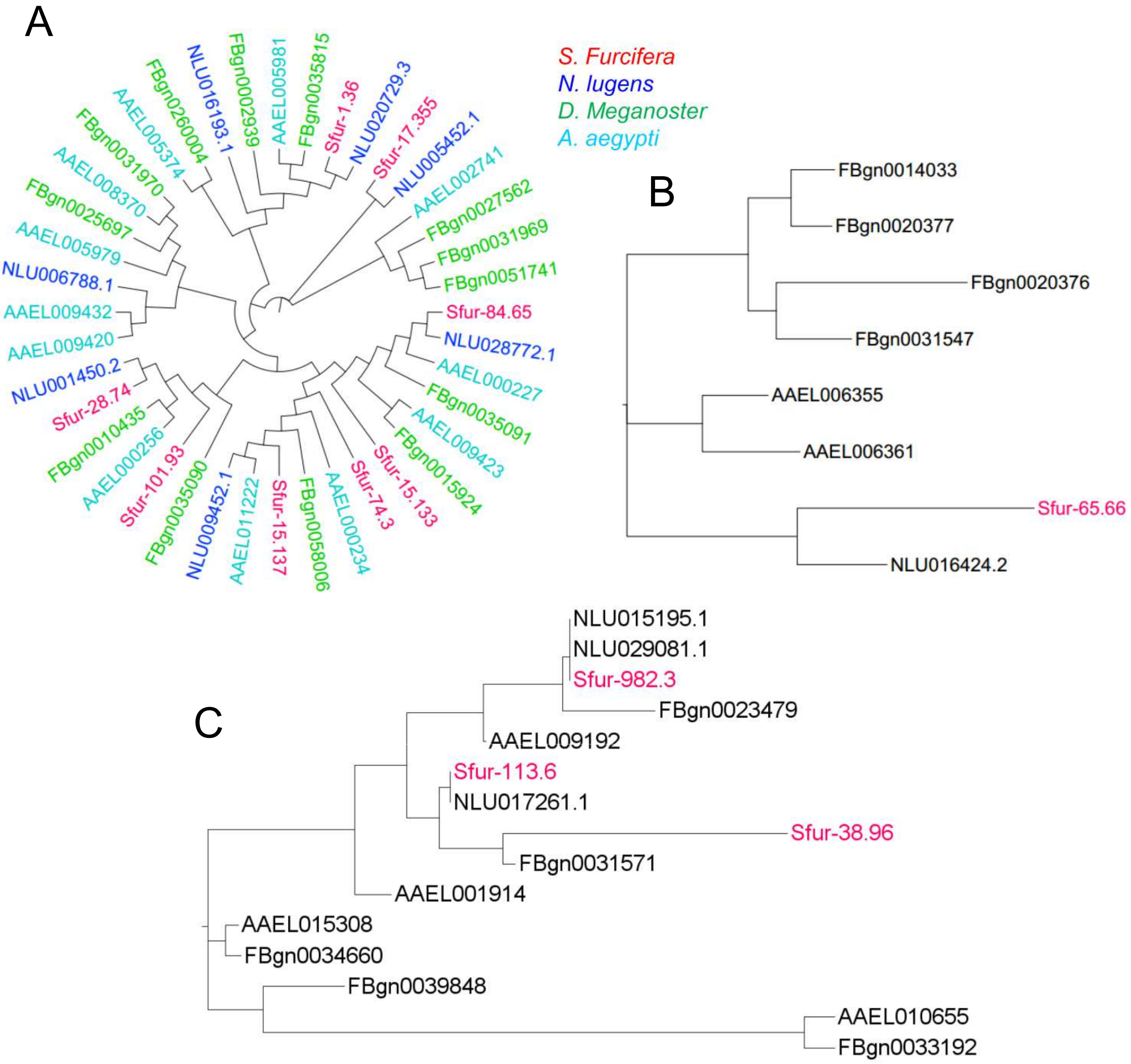

Figure S2. Phylogenetic analysis of scavenger receptors based on the sequences from four insects *S. furcifera, N. lugens, D. Melanogaster* and *A. aegypti*. (A). Phylogenetic analysis of SCRB. (B). Phylogenetic analysis of SCRC. (C). Phylogenetic analysis of SCRA.

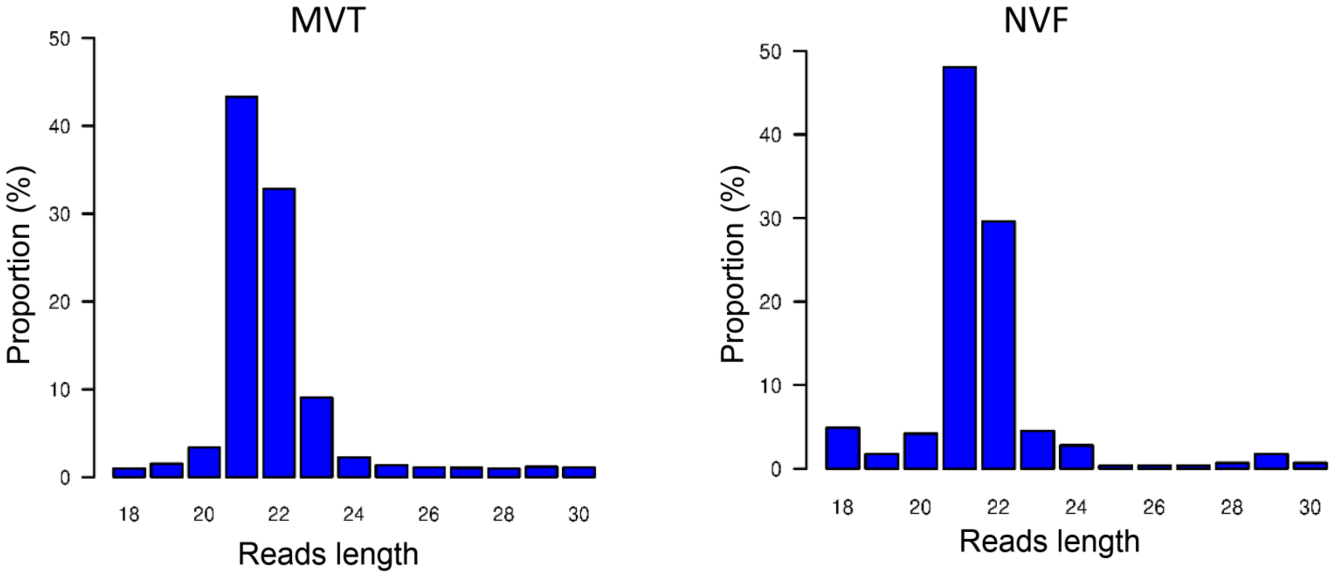

Figure S3. Length distribution of viral small RNAs in MVT and NVF libraries.
These reads were mapped on SRBSDV genome with 0-1 mismatch.

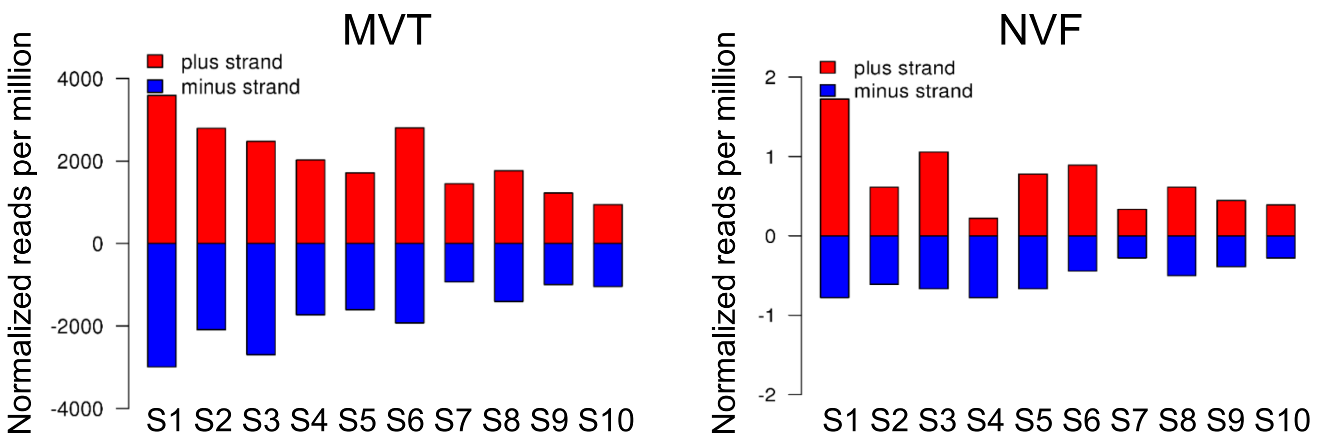

Figure S4. Distribution of viral small RNAs across SRBSDV segments. These reads were mapped on SRBSDV genome with 0-1 mismatch. Red bars represent reads mapping to the plus strand of the genome, blue bars depict reads from the minus strand.

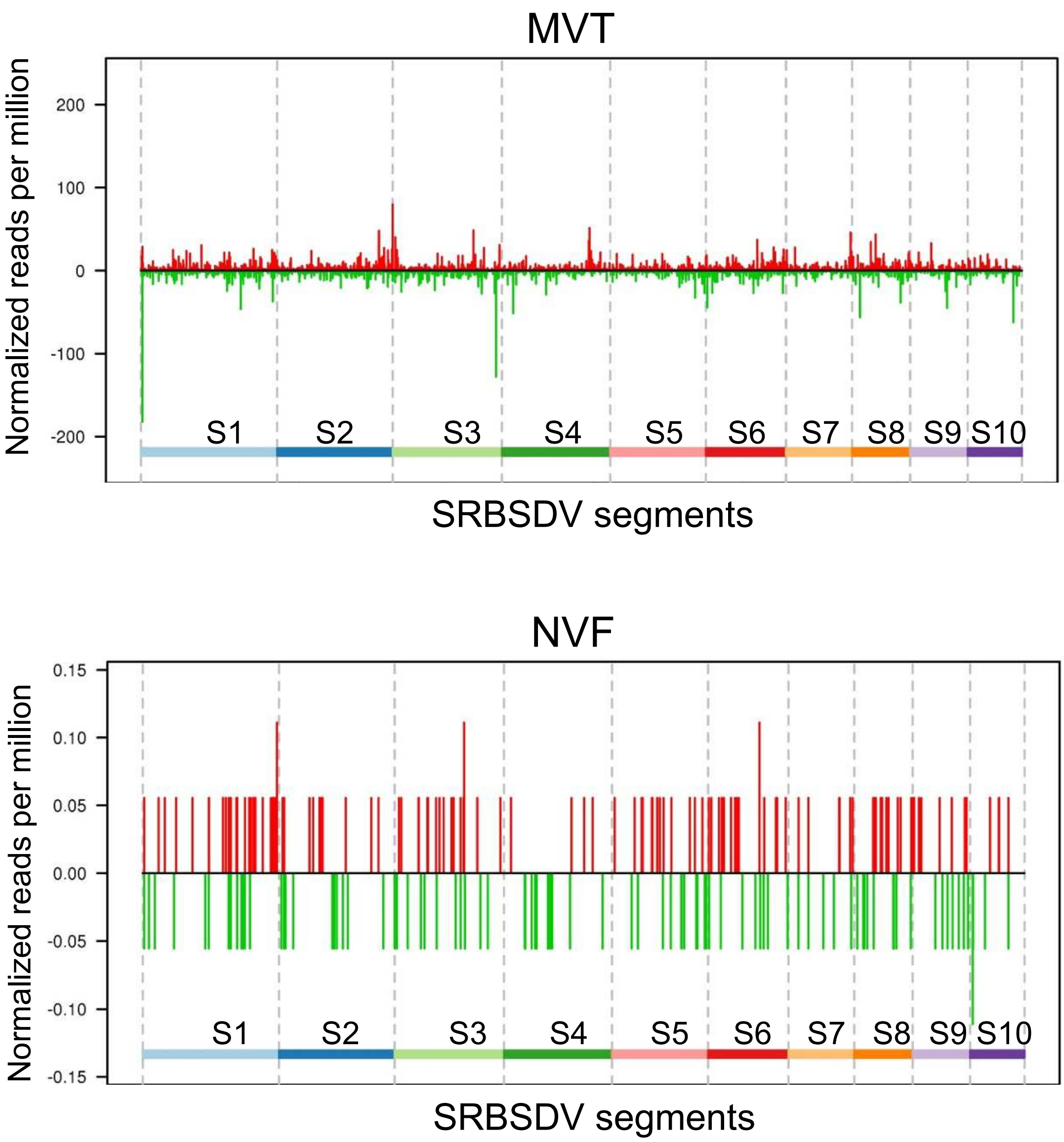

Figure S5. Distribution of 21-22 nt viral siRNAs across the SRBSDV segments. X-axis represents nucleotide positions of each SRBSDV segment. Y-axis represents the abundance of viral siRNAs derived from plus strand (in red) and minus strand (in green).

Table S1. The length and genomic location of *S. furcifera* immune-related genes

Gene families	Gene name	S. furcifera	Length (aa)	Position
RNA interference	AGO2	Sfur-72.600	1428	Scaffold-72:12085052:12144874:-
	Dcr-2	Sfur-139.28	2186	Scaffold-139:532985:590929:-
	r2d2	Sfur-20.248	514	Scaffold-20:6308822:6317874:+
	loqs	Sfur-50.248	767	Scaffold-50:2980448:3005829:-
	Hen1	Sfur-230.43	580	Scaffold-230:703662:716214:-
	drosha	Sfur-223.33	1470	Scaffold-223:591595:600005:-
	pasha	Sfur-504.10	825	Scaffold-504:189208:193788:+
	dicer-1	Sfur-151.9	2408	Scaffold-151:111942:156291:+
	AGO1	Sfur-63.27	941	Scaffold-63:496214:532159:+
	AGO3	Sfur-63.71	638	Scaffold-63:1412610:1447497:-
	translin	Sfur-450.9	217	Scaffold-450:123901:125043:+
		Sfur-148.30	244	Scaffold-148:444188:455617:-
	Fmr1	Sfur-172.36	699	Scaffold-172:749316:779876:-
	armitage	Sfur-326.2	1529	Scaffold-326:28748:68080:+
	spn-E	Sfur-513.9	1348	Scaffold-513:207469:263986:-
	CRIF	Sfur-6.74	255	Scaffold-6:1300928:1301945:+
	CG17265	Sfur-542.11	449	Scaffold-542:230231:236575:+
		Sfur-641.4	446	Scaffold-641:134980:141634:-
	Cbp80	Sfur-19.148	802	Scaffold-19:3422899:3478056:+
	Elp1	Sfur-72.23	2210	Scaffold-72:270020:304665:+
	Tis11	Sfur-267.17	304	Scaffold-267:322278:374007:-
	Ars2	Sfur-17.78	871	Scaffold-17:1515324:1554169:+
	aubergine	Sfur-22.29	949	Scaffold-22:864160:889166:-
	pcm	Sfur-503.9	1518	Scaffold-503:156893:202935:-
	bel	Sfur-72.131	1114	Scaffold-72:2169965:2212675:-
	hdc	Sfur-17.133	226	Scaffold-17:2809090:2809767:-
		Sfur-17.131	360	Scaffold-17:2720970:2787399:-
Toll signaling pathway	cact	Sfur-184.26	636	Scaffold-184:477522:494628:+
	Myd88	Sfur-169.16	189	Scaffold-169:446528:450576:+
	Pelle	Sfur-95.27	1992	Scaffold-95:430748:481309:-
	spz	Sfur-54.115	566	Scaffold-54:2058372:2079173:+
	spz3	Sfur-24.293	390	Scaffold-24:7169425:7216883:+
	spz4	Sfur-33.150	507	Scaffold-33:2533097:2541457:-
	spz5	Sfur-261.1	1830	Scaffold-261:1218:105685:+
	spz6	Sfur-5.212	507	Scaffold-5:4596404:4604268:-
	Tl	Sfur-33.32	1079	Scaffold-33:381269:392154:+
	Dorsal	Sfur-213.33	1324	Scaffold-213:416851:449531:+
	dif	Sfur-152.4	707	Scaffold-152:37993:59581:-
	tollip	Sfur-1.116	283	Scaffold-1:2070877:2077910:-

	tube	Sfur-23.126	502	Scaffold-23:2433405:2457027:+
	Hel89B	Sfur-72.337	1825	Scaffold-72:6253603:6295642:-
	Pli	Sfur-141.21	405	Scaffold-141:391115:409284:-
	Ulp1	Sfur-374.8	217	Scaffold-374:99750:105290:-
	mop	Sfur-120.31	2042	Scaffold-120:719241:769863:-
		Sfur-222.1	263	Scaffold-222:635:5899:-
	puf	Sfur-76.16	3399	Scaffold-76:399715:465615:-
	krz	Sfur-223.13	812	Scaffold-223:136074:213053:-
	Gprk2	Sfur-20.8	658	Scaffold-20:91299:110624:-
IMD signaling pathway	caspar	Sfur-17.324	640	Scaffold-17:7041532:7068673:-
		Sfur-150.27	909	Scaffold-150:508354:534321:+
	Diap1	Sfur-529.12	592	Scaffold-529:214043:230293:+
	IKKgamma(key)	Sfur-13.59	736	Scaffold-13:810071:814236:-
	IKKepsilon	Sfur-410.14	1705	Scaffold-410:302488:373452:+
	imd	Sfur-17.218	250	Scaffold-17:4620946:4627921:+
	Rel	Sfur-1444.20	2434	Scaffold-1444:377656:459802:+
	Tab2	Sfur-186.23	374	Scaffold-186:376453:386186:+
	Tak1	Sfur-9.184	459	Scaffold-9:3264533:3282901:+
	Dredd	Sfur-460.16	658	Scaffold-460:288818:301975:+
	Traf-like	Sfur-17.342	510	Scaffold-17:7444785:7448848:+
	Traf4	Sfur-66.92	454	Scaffold-66:1752324:1781555:+
	Ubc13	Sfur-251.194	152	Scaffold-251:2376907:2378585:+
	TM9SF2	Sfur-184.28	635	Scaffold-184:528926:545514:-
		Sfur-396.5	662	Scaffold-396:95424:112169:+
	TM9SF4	Sfur-70.81	628	Scaffold-70:1939943:1955965:-
JAK-STAT cascade	dome	Sfur-27.95	1146	Scaffold-27:2056152:2110712:+
	hop	Sfur-24.235	1149	Scaffold-24:6194992:6212334:+
	Stat92E	Sfur-86.26	819	Scaffold-86:538648:574609:+
	Su(var)2-10	Sfur-38.48	1346	Scaffold-38:631139:683688:-
	Socs36E	Sfur-101.37	742	Scaffold-101:433811:453034:+
		Sfur-28.3	634	Scaffold-28:27728:38467:+
		Sfur-315.10	498	Scaffold-315:303506:305470:+
	Socs16D	Sfur-289.13	536	Scaffold-289:259475:275878:-
	Socs44A	Sfur-64.68	405	Scaffold-64:1846456:1847958:-
	E(bx)	Sfur-40.46	2809	Scaffold-40:840470:903746:-
	kn	Sfur-114.36	555	Scaffold-114:584316:693007:+
	asrij	Sfur-51.78	467	Scaffold-51:1847845:1856507:-
	mask	Sfur-161.6	2812	Scaffold-161:199510:268864:-
melanization defense response	PPO	Sfur-106.2	1519	Scaffold-106:22332:80613:+
		Sfur-283.14	674	Scaffold-283:250627:263761:-
		Sfur-62.181	693	Scaffold-62:2689850:2708591:+
	serpin	Sfur-204.21	396	Scaffold-204:469759:487164:-

		Sfur-335.4	1580	Scaffold-335:52395:70236:+
		Sfur-350.3	541	Scaffold-350:94048:100689:-
		Sfur-377.2	1130	Scaffold-377:20825:57190:-
		Sfur-45.55	422	Scaffold-45:1207498:1233798:+
		Sfur-470.6	902	Scaffold-470:94763:113675:-
		Sfur-2570.1	144	Scaffold-2570:521:9244:+
		Sfur-71.69	839	Scaffold-71:1251056:1271974:-
		Sfur-88.14	599	Scaffold-88:238566:256328:-
		Sfur-97.15	283	Scaffold-97:281314:286609:+
	CLIP-domain serine protease	Sfur-364.6	725	Scaffold-364:117445:141627:-
		Sfur-389.3	674	Scaffold-389:31857:88292:-
		Sfur-745.2	773	Scaffold-745:67730:84072:+
		Sfur-49.2	1600	Scaffold-49:86919:138347:+
		Sfur-234.17	393	Scaffold-234:414952:424743:-
		Sfur-54.75	569	Scaffold-54:1485881:1504889:+
		Sfur-9.240	542	Scaffold-9:4261579:4288294:-
	bsk	Sfur-95.52	722	Scaffold-95:940615:1011164:-
	egr	Sfur-88.72	381	Scaffold-88:1406853:1451622:+
	hep	Sfur-105.58	1468	Scaffold-105:1080031:1152391:+
	Rho1	Sfur-15.272	193	Scaffold-15:6382420:6398542:-
		Sfur-39.58	198	Scaffold-39:1396821:1400937:+
pattern recognition receptor	C-lectin	Sfur-115.19	1399	Scaffold-115:395000:437892:+
		Sfur-1233.4	87	Scaffold-1233:24696:25315:-
		Sfur-135.14	284	Scaffold-135:155503:164681:-
		Sfur-7.132	1220	Scaffold-7:3368036:3409056:+
		Sfur-1591.2	377	Scaffold-1591:14026:22754:-
		Sfur-20.43	210	Scaffold-20:1250883:1255841:+
		Sfur-276.9	226	Scaffold-276:206934:228516:+
		Sfur-925.2	207	Scaffold-925:54977:59363:+
		Sfur-635.1	1222	Scaffold-635:130696:171774:+
		Sfur-308.14	2533	Scaffold-308:357433:462847:-
		Sfur-438.7	774	Scaffold-438:180639:295948:-
	GNBP	Sfur-168.43	1729	Scaffold-168:735156:778444:-
		Sfur-449.5	2161	Scaffold-449:123216:177396:+
		Sfur-186.24	542	Scaffold-186:388922:408037:+
		Sfur-71.64	1205	Scaffold-71:1083269:1138287:+
	PGRP-LC	Sfur-122.48	1364	Scaffold-122:1078566:1117556:+
	PGRP-LB	Sfur-20.196	499	Scaffold-20:5382138:5388174:-
	Draper	Sfur-78.16	944	Scaffold-78:344988:407245:-
	dscam	Sfur-115.24	14063	Scaffold-115:540151:720943:-
		Sfur-160.34	1636	Scaffold-160:643330:739246:+
		Sfur-17.263	2171	Scaffold-17:5671259:5736018:-
		Sfur-205.33	3270	Scaffold-205:546848:690961:+

		Sfur-413.23	1301	Scaffold-413:376783:437386:+
		Sfur-79.65	1860	Scaffold-79:1228387:1280718:+
	Eater	Sfur-13.168	1618	Scaffold-13:2870923:2897529:+
	galectin	Sfur-133.5	1153	Scaffold-133:124471:166675:-
		Sfur-169.42	2011	Scaffold-169:789485:872547:-
		Sfur-9.77	1312	Scaffold-9:1295870:1322459:+
	hemocytin	Sfur-199.20	4409	Scaffold-199:448045:536423:-
		Sfur-769.5	3557	Scaffold-769:81176:118211:+
defense response to virus	Atg18a	Sfur-78.63	455	Scaffold-78:1262805:1294285:+
	Atg7	Sfur-146.38	366	Scaffold-146:731158:735420:+
		Sfur-602.5	593	Scaffold-602:68037:77373:+
	CG10333	Sfur-347.22	653	Scaffold-347:400887:410359:-
	me31B	Sfur-169.35	449	Scaffold-169:687218:700687:+
	Mt2	Sfur-3.151	331	Scaffold-3:2892126:2904582:+
	Toll-7	Sfur-11.171	1323	Scaffold-11:3191439:3195849:+
	Toll-6	Sfur-176.31	1288	Scaffold-176:731355:735465:+
	Toll-8	Sfur-29.104	1721	Scaffold-29:1823673:1855334:-
	Toll-10	Sfur-230.19	1296	Scaffold-230:225496:259339:-
	Toll-13	Sfur-122.298	866	Scaffold-122:5156187:5168119:-
	FoxK	Sfur-72.391	552	Scaffold-72:7390551:7418684:-
	G9a	Sfur-11.142	1375	Scaffold-11:2817660:2844784:-
antimicrobial humoral response	alphaTub84B	Sfur-114.27	450	Scaffold-114:390989:395994:-
		Sfur-45.44	856	Scaffold-45:908888:927508:+
		Sfur-450.13	504	Scaffold-450:174663:179960:+
		Sfur-22.63	451	Scaffold-22:1702506:1744788:-
		Sfur-214.43	808	Scaffold-214:740479:759618:-
		Sfur-9.290	685	Scaffold-9:5208697:5237177:+
	Dlg5	Sfur-33.149	1937	Scaffold-33:2476623:2525174:+
	dos	Sfur-19.265	668	Scaffold-19:5424741:5445662:+
	Eip75B	Sfur-40.72	942	Scaffold-40:1654489:1681434:+
	lola	Sfur-124.39	355	Scaffold-124:859866:869751:-
	lysozyme	Sfur-11.139	154	Scaffold-11:2779444:2782951:-
		Sfur-145.15	307	Scaffold-145:293700:304175:-
		Sfur-53.22	174	Scaffold-53:313172:333372:+
		Sfur-85.10	278	Scaffold-85:229207:239997:+
		Sfur-85.9	156	Scaffold-85:223212:227637:+
	mbo	Sfur-7.118	501	Scaffold-7:3076245:3112006:+
	Mef2	Sfur-35.38	709	Scaffold-35:554076:603181:+
	Ntf-2	Sfur-9.95	131	Scaffold-9:1506325:1512416:+
	par-1	Sfur-14.79	579	Scaffold-14:1657393:1695616:+
	Parp	Sfur-72.107	1092	Scaffold-72:1769730:1789955:-
	PCNA	Sfur-74.129	261	Scaffold-74:1594683:1599801:-
	smog	Sfur-56.66	675	Scaffold-56:1559920:1575568:-

	Ptip	Sfur-49.48	1425	Scaffold-49:1229167:1288615:+
	Pvr	Sfur-27.55	2106	Scaffold-27:917936:947832:-
		Sfur-170.3	818	Scaffold-170:25331:45602:+
	Rab11	Sfur-13.193	216	Scaffold-13:3151181:3168837:+
	scrib	Sfur-347.18	1837	Scaffold-347:240129:358804:+
	Spt6	Sfur-98.17	1806	Scaffold-98:343206:382860:-
	Thor	Sfur-207.15	119	Scaffold-207:346434:353919:+
	Tlk	Sfur-682.3	933	Scaffold-682:17219:73518:+
	Vps15	Sfur-270.24	1339	Scaffold-270:459835:498283:+
	vvl	Sfur-250.11	433	Scaffold-250:190874:192196:-
	zfh1	Sfur-36.98	912	Scaffold-36:2026389:2055531:-
antimicrobial peptide	Defensin	Sfur-10.120	340	Scaffold-10:2352765:2370451:+
		Sfur-33.51	262	Scaffold-33:660700:668230:+
	NOS	Sfur-9.52	1383	Scaffold-9:663560:726000:-
encapsulation of foreign target	alpha-Man-Ia	Sfur-17.159	525	Scaffold-17:3405714:3418388:-
	alpha-Man-Ib	Sfur-241.29	834	Scaffold-241:636578:661827:+
		Sfur-489.8	585	Scaffold-489:254802:271669:-
	alpha-Man-IIa	Sfur-39.3	1116	Scaffold-39:15322:46715:-
	alpha-Man-II b	Sfur-129.28	1650	Scaffold-129:429695:442551:-
		Sfur-825.7	588	Scaffold-825:85354:94586:+
		Sfur-70.20	1030	Scaffold-70:239001:296085:+
	Als2	Sfur-494.2	1647	Scaffold-494:26614:63132:-
	aPKC	Sfur-116.56	597	Scaffold-116:1045903:1097143:+
	Cdc42	Sfur-171.14	650	Scaffold-171:343914:368853:+
	Cdk5	Sfur-50.56	303	Scaffold-50:739988:745845:-
	Cdk5alpha	Sfur-150.22	282	Scaffold-150:434390:435235:-
		Sfur-150.19	213	Scaffold-150:407613:409827:-
	DCTN1-p150	Sfur-85.11	1283	Scaffold-85:241112:278049:-
	DCX-EMAP	Sfur-518.5	410	Scaffold-518:98833:107254:+
	Dhc64C	Sfur-63.19	4660	Scaffold-63:296196:374130:-
	dia	Sfur-20.194	1060	Scaffold-20:5305796:5347302:+
	Eph	Sfur-18.207	835	Scaffold-18:3960077:4010736:-
	Ephrin	Sfur-15.173	554	Scaffold-15:4094700:4142237:+
	Exn	Sfur-877.3	1999	Scaffold-877:14368:47954:+
	Flo2	Sfur-229.21	425	Scaffold-229:370218:371492:-
	LanA	Sfur-359.5	3683	Scaffold-359:105895:174267:+
	OstDelta	Sfur-223.18	1578	Scaffold-223:267985:291271:+
	Ostgamma	Sfur-324.8	699	Scaffold-324:181936:222244:-
	RhoBTB	Sfur-33.154	1331	Scaffold-33:2587153:2633646:-
	RhoGEF3	Sfur-18.90	1075	Scaffold-18:1959210:1997732:-
	Shc	Sfur-11755.1	153	Scaffold-11755:17:677:-
		Sfur-54.85	597	Scaffold-54:1579537:1581507:-
		Sfur-54.99	1176	Scaffold-54:1742096:1780551:-

	Vav	Sfur-23.69	1713	Scaffold-23:1307574:1338992:-
	Zir	Sfur-1433.1	1661	Scaffold-1433:28:24184:-
		Sfur-440.5	2606	Scaffold-440:306742:330262:+
phagocytosis	crq	Sfur-51.4	1564	Scaffold-51:42730:124978:+
		Sfur-13.116	547	Scaffold-13:1948339:1978094:+
	brm	Sfur-108.44	1476	Scaffold-108:763820:783750:-
		Sfur-598.8	1267	Scaffold-598:148332:179894:+
	EcR	Sfur-50.12	619	Scaffold-50:203218:222293:-
	pnt	Sfur-57.78	392	Scaffold-57:1777646:1805416:-
	betaCOP	Sfur-431.10	916	Scaffold-431:152224:163621:+
	gcm	Sfur-28.6	572	Scaffold-28:112031:121193:-
		Sfur-84.70	629	Scaffold-84:1328003:1344380:-
	Trn	Sfur-69.5	929	Scaffold-69:126622:205028:+
	DMAP1	Sfur-85.55	2735	Scaffold-85:981301:1019407:+
	Nup98-96	Sfur-16.163	1988	Scaffold-16:3378124:3436367:+
		Sfur-1672.1	572	Scaffold-1672:1280:19310:+
		Sfur-1251.1	253	Scaffold-1251:6001:18296:+
	kuz	Sfur-192.21	789	Scaffold-192:451807:476216:+
mucosal immune response	bbg	Sfur-38.58	1436	Scaffold-38:897161:916430:-
	Duox	Sfur-829.6	816	Scaffold-829:73630:99301:-
		Sfur-921.1	422	Scaffold-921:5453:14209:+
	Galphaq	Sfur-72.236	354	Scaffold-72:3794880:3840867:-
	lic	Sfur-14.118	331	Scaffold-14:2407999:2417460:+
	Mekk1	Sfur-17.172	1315	Scaffold-17:3644468:3679361:-
	Mkp3	Sfur-67.335	662	Scaffold-67:4917358:4947390:+
	norpA	Sfur-268.14	1904	Scaffold-268:197055:265330:-
		Sfur-68.11	1079	Scaffold-68:201289:241336:+
		Sfur-226.20	1202	Scaffold-226:343583:379944:+
	p38	Sfur-17.307	622	Scaffold-17:6675902:6707748:+
negative regulation of immune response	Atg6	Sfur-596.2	373	Scaffold-596:22962:36274:-
	dnr1	Sfur-20.247	883	Scaffold-20:6267460:6306119:-
	Dsp1	Sfur-246.15	210	Scaffold-246:252378:274923:-
		Sfur-246.14	1047	Scaffold-246:220688:247583:-
	faf	Sfur-8.174	2764	Scaffold-8:3190206:3253519:+
	GlyP	Sfur-487.5	1675	Scaffold-487:130966:195898:+
	Lam	Sfur-197.34	549	Scaffold-197:575614:600981:+
	MED6	Sfur-295.17	214	Scaffold-295:294849:302320:+
		Sfur-951.1	234	Scaffold-951:46501:56132:-
	Prx5	Sfur-68.55	818	Scaffold-68:1213546:1255072:-
	Rala	Sfur-727.2	308	Scaffold-727:65697:77873:+
	RYBP	Sfur-7.142	669	Scaffold-7:3890807:3912355:-
	sax	Sfur-20.106	651	Scaffold-20:3024141:3078538:+
	scny	Sfur-84.32	1041	Scaffold-84:431035:455442:+

	slif	Sfur-230.42	1500	Scaffold-230:665771:699639:-
		Sfur-50.193	727	Scaffold-50:2444004:2475761:+
		Sfur-345.27	957	Scaffold-345:420402:455888:+
	SP2353	Sfur-197.44	308	Scaffold-197:788821:808743:+
	tefu	Sfur-158.19	2853	Scaffold-158:190732:276365:+
	Tim10	Sfur-338.15	96	Scaffold-338:290741:295170:+
	unc-45	Sfur-97.14	946	Scaffold-97:277855:280692:+
	Usp2	Sfur-20.70	517	Scaffold-20:2065496:2082524:+
	Nrf2	Sfur-9.282	637	Scaffold-9:4962381:5019134:-
	wun	Sfur-273.10	279	Scaffold-273:78819:104116:+
positive regulation of immune response	att-ORFA	Sfur-622.8	321	Scaffold-622:155464:169124:+
	CG2051	Sfur-16.19	411	Scaffold-16:349132:369417:-
	CG32795	Sfur-32.48	646	Scaffold-32:380915:398262:+
	chico	Sfur-77.62	1062	Scaffold-77:999570:1011562:-
	Crag	Sfur-89.92	1869	Scaffold-89:1344519:1427737:-
	Etl1	Sfur-181.1	1542	Scaffold-181:1329:26514:+
	HBS1	Sfur-134.8	1321	Scaffold-134:193802:219154:-
	Hr78	Sfur-162.14	656	Scaffold-162:279768:292433:+
	htl	Sfur-27.19	1019	Scaffold-27:447830:489575:-
	Ide	Sfur-36.56	967	Scaffold-36:1028818:1051118:+
	kug	Sfur-369.15	3149	Scaffold-369:201228:270273:+
		Sfur-316.19	2937	Scaffold-316:361388:448023:+
		Sfur-6.221	3111	Scaffold-6:3682324:3750622:+
		Sfur-316.13	612	Scaffold-316:221741:223576:+
		Sfur-6.194	1141	Scaffold-6:3372268:3375690:+
	LanB1	Sfur-24.172	1752	Scaffold-24:4571900:4606027:+
	Mmp2	Sfur-259.18	494	Scaffold-259:381746:394957:+
	mtd	Sfur-8.143	960	Scaffold-8:2391353:2526890:-
	PDCD-5	Sfur-169.12	130	Scaffold-169:413640:417815:-
	puc	Sfur-167.52	266	Scaffold-167:758620:801656:+
	Sec31	Sfur-11.25	1849	Scaffold-11:621923:686736:+
immune response	Atf3	Sfur-125.49	525	Scaffold-125:908975:918974:+
	cher	Sfur-7.140	2355	Scaffold-7:3611650:3797156:+
	CSN5	Sfur-44.49	345	Scaffold-44:814419:818993:-
	Deaf1	Sfur-438.6	500	Scaffold-438:156032:178237:+
	eya	Sfur-20.138	776	Scaffold-20:3947459:3975228:+
	modSP	Sfur-102.37	1173	Scaffold-102:890003:915418:+
		Sfur-79.44	712	Scaffold-79:726696:746148:-
	Reeler	Sfur-159.27	270	Scaffold-159:455725:466829:+
	Uba2	Sfur-594.3	642	Scaffold-594:33778:53907:-
		Sfur-691.11	577	Scaffold-691:147456:160638:-
	Vps16B	Sfur-97.22	440	Scaffold-97:493395:503004:+
	Vps33B	Sfur-107.10	738	Scaffold-107:221949:231075:-

Caspase	Caspase	Sfur-17.341	291	Scaffold-17:7425599:7442532:+
	Caspase	Sfur-6.178	1178	Scaffold-6:3076669:3121666:+
	Caspase	Sfur-1194.2	602	Scaffold-1194:25502:35039:-
	Caspase	Sfur-23.136	781	Scaffold-23:2651782:2681095:-
	Caspase	Sfur-739.1	598	Scaffold-739:13669:21225:+
	Caspase	Sfur-460.16	658	Scaffold-460:288818:301975:+
	Caspase	Sfur-24.353	793	Scaffold-24:8701529:8717817:+
Catalase	Catalase	Sfur-314.12	504	Scaffold-314:185421:217844:-
Fibrinogen-like Proteins	Fibrinogen-like Proteins	Sfur-418.10	746	Scaffold-418:136687:164330:-
	Fibrinogen-like Proteins	Sfur-72.247	1931	Scaffold-72:3955028:3966237:+
IAP Repeat	IAP Repeat	Sfur-234.14	4551	Scaffold-234:284350:376956:+
	IAP Repeat	Sfur-119.29	235	Scaffold-119:602787:617554:-
MD-2-related lipid recognation	MD-2-related lipid recognation	Sfur-283.24	172	Scaffold-283:474795:478468:+
Scavenger Receptiors	Scavenger Receptiors	Sfur-28.74	911	Scaffold-28:1397713:1437306:+
	Scavenger Receptiors	Sfur-65.66	1310	Scaffold-65:1462168:1501916:+
	Scavenger Receptiors	Sfur-101.93	931	Scaffold-101:1324443:1379975:-
	Scavenger Receptiors	Sfur-84.65	198	Scaffold-84:1250921:1256071:+
	Scavenger Receptiors	Sfur-38.96	2869	Scaffold-38:1336839:1445976:+
	Scavenger Receptiors	Sfur-982.3	611	Scaffold-982:43664:55543:-
	Scavenger Receptiors	Sfur-113.6	3665	Scaffold-113:75649:181553:+
	Scavenger Receptiors	Sfur-74.3	467	Scaffold-74:32128:58667:-
	Scavenger Receptiors	Sfur-168.24	1040	Scaffold-168:469889:496900:-
	Scavenger Receptiors	Sfur-17.355	596	Scaffold-17:7698657:7718981:+
	Scavenger Receptiors	Sfur-15.137	586	Scaffold-15:3005222:3080960:+
	Scavenger Receptiors	Sfur-1.36	507	Scaffold-1:740692:752444:+
	Scavenger Receptiors	Sfur-15.133	1136	Scaffold-15:2944056:2982430:+

Spatzle	Spatzle	Sfur-54.115	566	Scaffold-54:2058372:2079173:+
	Spatzle	Sfur-5.212	507	Scaffold-5:4596404:4604268:-
	Spatzle	Sfur-24.293	390	Scaffold-24:7169425:7216883:+
	Spatzle	Sfur-166.29	436	Scaffold-166:571154:592970:+
	Spatzle	Sfur-261.1	1830	Scaffold-261:1218:105685:+
	Spatzle	Sfur-72.508	541	Scaffold-72:10049305:10066841:-
	Spatzle	Sfur-33.150	507	Scaffold-33:2533097:2541457:-
Superoxide dismutase	Superoxide dismutase	Sfur-13.25	930	Scaffold-13:297893:301420:-
	Superoxide dismutase	Sfur-41.88	2235	Scaffold-41:2344888:2360466:+
	Superoxide dismutase	Sfur-8.79	1684	Scaffold-8:1073640:1081782:-
	Superoxide dismutase	Sfur-23.71	349	Scaffold-23:1354194:1372952:-
	Superoxide dismutase	Sfur-33.90	465	Scaffold-33:1452813:1478112:-
	Superoxide dismutase	Sfur-67.126	561	Scaffold-67:1680925:1698744:-
	Superoxide dismutase	Sfur-67.143	348	Scaffold-67:1838161:1869273:-
Thioester-containing protein	Thioester-containing protein	Sfur-70.51	2555	Scaffold-70:831849:919436:+
peroxidase	peroxidase	Sfur-219.18	1120	Scaffold-219:248013:300149:+
	peroxidase	Sfur-126.46	1216	Scaffold-126:746464:797465:-
	peroxidase	Sfur-46.51	2353	Scaffold-46:1175066:1240084:-
	peroxidase	Sfur-77.96	1362	Scaffold-77:1494315:1530461:-
	peroxidase	Sfur-18.30	576	Scaffold-18:655849:697622:-
	peroxidase	Sfur-120.36	2359	Scaffold-120:852675:919715:+
	peroxidase	Sfur-273.7	234	Scaffold-273:62169:73061:+
	peroxidase	Sfur-47.125	2918	Scaffold-47:2235742:2293595:-
	peroxidase	Sfur-30.107	1499	Scaffold-30:2304735:2336216:+
	peroxidase	Sfur-15.277	199	Scaffold-15:6434239:6443533:-
	peroxidase	Sfur-65.41	1425	Scaffold-65:860460:898413:+
	peroxidase	Sfur-17.242	3270	Scaffold-17:5223007:5297678:+
	peroxidase	Sfur-430.8	409	Scaffold-430:169988:185780:-
	peroxidase	Sfur-6.74	255	Scaffold-6:1300928:1301945:+
	peroxidase	Sfur-22.68	265	Scaffold-22:1849864:1857820:-
	peroxidase	Sfur-128.19	221	Scaffold-128:569294:576632:+
	peroxidase	Sfur-22.79	184	Scaffold-22:2064821:2069885:-

Note: The detailed gene sequence will be available on the link http://staff.ustc.edu.cn/~wuqf/resources.html

Table S2. Classification of immune related genes of *S. furcifera, N. lugens* and *D. Melanogaster*

category	gene name	S. furcifera	N. lugens	D. melanogaster
RNA interference	AGO2	S.fur-72.600	NLU002433.1	FBgn0087035
	Dcr-2	S.fur-139.28	NLU028676.1	FBgn0034246
	r2d2	S.fur-20.248	NLU009200.1	FBgn0031951
	loqs	S.fur-50.248	NLU021888.1	FBgn0032515
	Hen1	S.fur-230.43	NLU016076.1,NLU019297.1,NLU016075.1	FBgn0033686
	drosha	S.fur-223.33	NLU024074.1	FBgn0026722
	pasha	S.fur-504.10	NLU019062.1,NLU025792.1	FBgn0039861
	dicer-1	S.fur-151.9	NLU006786.1	FBgn0039016
	AGO1	S.fur-63.27	NLU002737.1	FBgn0262739
	AGO3	S.fur-63.71	NLU019917.2	FBgn0250816
	translin	S.fur-450.9	/	/
		S.fur-148.30		
	Fmr1	S.fur-172.36	NLU028700.1	FBgn0028734
	armitage	S.fur-326.2	NLU005908.1	FBgn0041164
	spn-E	S.fur-513.9	NLU012389.1	FBgn0003483
	CRIF	S.fur-6.74	NLU004434.1	FBgn0037102
	CG17265	S.fur-542.11	NLU010721.1,NLU016057.1	FBgn0031488
		S.fur-641.4		
	Cbp80	S.fur-19.148	NLU019101.1	FBgn0022942

	Cbp20	/	NLU017365.1	FBgn0022943
	Elp1	S.fur-72.23	/	FBgn0037926
	Tis11	S.fur-267.17	NLU026267.1	FBgn0011837
	Ars2	S.fur-17.78	NLU025386.1	FBgn0033062
	blanks	/	/	FBgn0035608,<br>FBgn0035571
	piwi	/	NLU020171.1	FBgn0004872
	aubergine	S.fur-22.29	NLU014209.1	FBgn0000146
	ppk29	/	/	FBgn0034965
	pcm	S.fur-503.9	NLU016288.1	FBgn0020261
	bel	S.fur-72.131	NLU014452.1	FBgn0263231
	hdc	S.fur-17.133	NLU003528.1,NLU003527.1	FBgn0010113
		S.fur-17.131		
Toll signaling pathway	cact	S.fur-184.26	NLU007600.1	FBgn0000250
	Myd88	S.fur-169.16	NLU015637.1,NLU015640.1	FBgn0033402
	Pelle	S.fur-95.27	NLU007825.1	FBgn0010441
	spz	S.fur-54.115	NLU023813.1	FBgn0003495
	spz2	/	/	FBgn0261526
	spz3	S.fur-24.293	NLU021391.1,NLU019692.1	FBgn0031959
	spz4	S.fur-33.150	NLU006163.1	FBgn0032362
	spz5	S.fur-261.1	NLU021217.1	FBgn0035379
	spz6	S.fur-5.212	NLU021599.1,NLU021596.1	FBgn0035056
	Tl	S.fur-33.32	NLU007992.1,NLU025956.1	FBgn0262473
	Dorsal	S.fur-213.33	NLU004660.1	FBgn0260632

	Dif	S.fur-152.4	NLU001982.1	FBgn0011274
	tollip	S.fur-1.116	NLU024621.1	/
	tube	S.fur-23.126	/	FBgn0003882
	lwr	/	NLU009395.1	FBgn0010602
	Hel89B	S.fur-72.337	NLU002799.1	FBgn0022787
	Pli	S.fur-141.21	NLU008334.3	FBgn0025574
	Ulp1	S.fur-374.8	/	FBgn0027603
	mop	S.fur-120.31	NLU007526.1	FBgn0036448
		S.fur-222.1		
	puf	S.fur-76.16	NLU015336.1,NLU015334.1	FBgn0039214
	krz	S.fur-223.13	NLU028087.2	FBgn0040206
	Gprk2	S.fur-20.8	NLU001194.1	FBgn0261988
IMD signaling pathway	caspar	S.fur-17.324	NLU013036.1,NLU017992.1	FBgn0034068
		S.fur-150.27	NLU019739.1	/
		/	/	FBgn0025608
	Diap2	/	NLU021557.1,NLU021593.1	FBgn0015247
	Diap1	S.fur-529.12	NLU002427.1,NLU025325.1	FBgn0260635
	IKKbeta	/	NLU025262.1	FBgn0024222
	IKKgamma(key)	S.fur-13.59	/	FBgn0041205
	IKKepsilon	S.fur-410.14	NLU019937.1	FBgn0086657
	imd	S.fur-17.218	NLU011186.1	FBgn0013983
	Rel	S.fur-1444.20	NLU002823.1	FBgn0014018
	Tab2	S.fur-186.23	NLU006103.1	FBgn0086358
	Tak1	S.fur-9.184	NLU003382.2	FBgn0026323

	Dredd	S.fur-460.16	/	FBgn0020381
		/		
	Traf-like	S.fur-17.342	NLU005471.1	FBgn0030748
	Traf4	S.fur-66.92	NLU024884.1	FBgn0026319
	Traf6	/	NLU016291.2,NLU017152.1	FBgn0265464
	Ubc13	S.fur-251.194	NLU022111.1	FBgn0000173
	TM9SF2	S.fur-184.28	NLU010276.1	FBgn0032880
		S.fur-396.5		
	TM9SF4	S.fur-70.81	NLU013705.1,NLU024056.2	FBgn0028541
	Npc2a	/	/	FBgn0031381
	Npc2e	/	/	FBgn0051410
	pirk	/	/	FBgn0034647
JAK-STAT cascade	dome	S.fur-27.95	/	FBgn0043903
	hop	S.fur-24.235	NLU018705.1,NLU018707.1	FBgn0004864
	Stat92E	S.fur-86.26	NLU009978.1	FBgn0016917
	Su(var)2-10	S.fur-38.48	NLU002060.2	FBgn0003612
	upd1	/	/	FBgn0004956
	upd2	/	/	FBgn0030904
	upd3	/	/	FBgn0053542
	vir-1	/	/	FBgn0043841
	Socs36E	S.fur-101.37	NLU008288.1,NLU027078.1,NLU005783.1	FBgn0041184
		S.fur-28.3		
		S.fur-315.10		
	Socs16D	S.fur-289.13	NLU004891.1	FBgn0030869
	Socs44A	S.fur-64.68	NLU007597.1	FBgn0033266

	E(bx)	S.fur-40.46	NLU007175.1,NLU007172.1	FBgn0000541
	kn	S.fur-114.36	NLU011325.1	FBgn0001319
	asrij	S.fur-51.78	NLU017830.3	FBgn0034793
	mask	S.fur-161.6	NLU006840.1	FBgn0043884
melanization defense response	PPO	S.fur-106.2,S.fur-283.14,S.fur-62.181	NLU002909.1,NLU022227.1,NLU022111.1,NLU006547.1,NLU002910.1	FBgn0033367,FBgn0261363,FBgn0261362
	serpin	S.fur-204.21	NLU003320.1,NLU017458.1	/
		S.fur-335.4	NLU005641.1,NLU002506.1	FBgn0263109
		S.fur-350.3	NLU028092.1	FBgn0039795
		S.fur-377.2	NLU013115.1	FBgn0037772
		S.fur-45.55	NLU010255.1	FBgn0038299,FBgn0028984
		S.fur-470.6	NLU008425.1	FBgn0031973
		S.fur-2570.1		
		S.fur-71.69	NLU002705.1	/
		S.fur-88.14	NLU025039.1	/
		S.fur-97.15	/	/
		/	/	FBgn0028990
		/	/	FBgn0262057,FBgn0036969,FBgn0036970
		/	/	FBgn0015586,FBgn0052203
		/	/	/

		/	/	FBgn0028983,FBgn0028985,FBgn0033112,
		/	/	FBgn0002930,FBgn0032178,FBgn0044011,FBgn0024293,FBgn0024294,FBgn0034195,
		/	/	FBgn0028986,FBgn0028987,FBgn0028988,FBgn0083141,FBgn0051902,FBgn0053121,FBgn0033113,FBgn0033574,FBgn0033115
	CLIP-domain serine protease	S.fur-364.6	NLU015564.1,NLU008637.5	FBgn0038727,FBgn0036891
		S.fur-389.3		
		S.fur-745.2	NLU028830.1	FBgn0030927
		S.fur-49.2	NLU018819.2,NLU018820.1	FBgn0035501,FBgn0052260
		S.fur-234.17	NLU018349.1,NLU016731.1	FBgn0003450,FBgn0030051
		S.fur-54.75		
		S.fur-9.240		
		/	/	FBgn0085438
		/	/	FBgn0030925,FBgn0030926
		/	/	FBgn0039494,FBgn0039495

		/	/	FBgn0019929
		/	/	FBgn0039759, FBgn0039798
		/	/	FBgn0039758, FBgn0000533, FBgn0027930, FBgn0039102, FBgn0038250, FBgn0039101, FBgn0042106
		/	/	FBgn0051728
		/	/	/
		/	/	/
	bsk	S. fur-95. 52	NLU019825. 1	FBgn0000229
	Dark	/	/	FBgn0263864
	Dronc	/	/	FBgn0026404
	egr	S. fur-88. 72	/	FBgn0033483
	hep	S. fur-105. 58	NLU015503. 1	FBgn0010303
	Rho1	S. fur-15. 272	/	FBgn0014020
		S. fur-39. 58		
pattern recognition receptor	C-lectin	S. fur-115. 19	NLU015710. 1, NLU010305. 1	FBgn0001083
		S. fur-1233. 4	/	FBgn0035199
		S. fur-135. 14	NLU018769. 1	FBgn0038315
		S. fur-7. 132	NLU009485. 1	FBgn0037240, FBgn0034317, FBgn0034318
		S. fur-1591. 2	NLU005367. 1	FBgn0262720, FBgn0040502, FBgn0033067

		S. fur-20. 43	NLU018248. 1	FBgn0031629
		S. fur-276. 9		
		S. fur-925. 2	NLU016070. 1, NLU012907. 1	FBgn0031918
		S. fur-635. 1	NLU008455. 1	FBgn0038017
		S. fur-308. 14	NLU011397. 1, NLU025259. 1	FBgn0035031
		S. fur-438. 7	NLU009896. 1, NLU009897. 1	FBgn0030617
		/	/	FBgn0029814, FBgn0261258
		/	/	FBgn0053533, FBgn0040093, FBgn0040092, FBgn0030050, FBgn0262357, FBgn0259958, FBgn0053532, FBgn0016675, FBgn0040104, FBgn0264341, FBgn0259230, FBgn0040102, FBgn0040099, FBgn0031910, FBgn0031373, FBgn0040503, FBgn0040107, FBgn0040106, FBgn0031273, FBgn0040097, FBgn0040098, FBgn0015583
		/	/	FBgn0054033, FBgn0040096, FBgn0031879
		/	/	FBgn0031571, FBgn00327

				70
	GNBP	S.fur-168.43	NLU026651.1	/
		S.fur-449.5	NLU018508.1,NLU022390.1,NLU029010.1	/
		S.fur-186.24	NLU006102.1,NLU014039.2	FBgn0040322(GNBP2)
		S.fur-71.64		
		/	/	FBgn0040323(GNBP1)
		/	/	FBgn0040321(GNBP3)
		/	/	/
		/	/	/
	PGRP-LC	S.fur-122.48	NLU001564.1	FBgn0035976
	PGRP-LB	S.fur-20.196	NLU006527.1	FBgn0037906
	PGRP-LA	/	/	FBgn0035975
	PGRP-LD	/	/	FBgn0260458
	PGRP-LE	/	/	FBgn0030695
	PGRP-LF	/	/	FBgn0035977
	PGRP-SA	/	/	FBgn0030310
	PGRP-SB1	/	/	FBgn0043578
	PGRP-SB2	/	/	FBgn0043577
	PGRP-SC1a	/	/	FBgn0043576
	PGRP-SC1b	/	/	FBgn0033327
	PGRP-SC2	/	/	FBgn0043575
	PGRP-SD	/	/	FBgn0035806

	Draper	S.fur-78.16	NLU010296.1	FBgn0027594
	dscam	S.fur-115.24	NLU015558.1,NLU025113.1	/
		S.fur-160.34	NLU001230.1	/
		S.fur-17.263	NLU024552.1	/
		S.fur-205.33	NLU026279.1	/
		S.fur-413.23	NLU002721.1	FBgn0263218(Dscam2)
		S.fur-79.65	/	FBgn0263219(Dscam4)
		/	NLU012705.3	FBgn0033159(Dscam1)
		/	/	FBgn0261046(Dscam3)
		/	/	/
	Eater	S.fur-13.168	NLU028308.1	FBgn0243514
	galectin	S.fur-133.5	/	/
		S.fur-169.42	NLU004153.1	FBgn0031213,FBgn0031214
		S.fur-9.77	NLU014787.2	FBgn0052226
		/	/	FBgn0031289,FBgn0034365
		/	/	FBgn0038419
	hemocytin	S.fur-199.20	NLU018484.2	FBgn0029167
		S.fur-769.5		
defense response to virus	Atg18a	S.fur-78.63	NLU020875.1	FBgn0035850
	Atg7	S.fur-146.38	NLU009932.1	FBgn0034366
		S.fur-602.5		

	CG10333	S.fur-347.22	NLU014381.1	FBgn0032690
	CG12780	/	/	FBgn0033301
	me31B	S.fur-169.35	NLU015620.3	FBgn0004419
	Mt2	S.fur-3.151	/	FBgn0028707
	Toll-7	S.fur-11.171	NLU009021.1	FBgn0034476
	Toll-6	S.fur-176.31	NLU008392.1	FBgn0036494
	Toll-8	S.fur-29.104	NLU027363.1	FBgn0029114
	Toll-10	S.fur-230.19	NLU022764.1	/
	Toll-13	S.fur-122.298	NLU027922.1	/
	Toll-2	/	/	FBgn0004364
	Toll-3	/	/	FBgn0015770
	Toll-4	/	/	FBgn0032095
	Toll-5	/	/	FBgn0026760
	Toll-9	/	/	FBgn0036978
	DIP1	/	NLU014786.1	FBgn0024807
	FoxK	S.fur-72.391	NLU006150.1	FBgn0036134
	G9a	S.fur-11.142	NLU019099.2	FBgn0040372
antimicrobial humoral response	alphaTub84B	S.fur-114.27	NLU005428.1,NLU004930.1	/
		S.fur-45.44	NLU007674.1,NLU010638.1	/
		S.fur-450.13	/	FBgn0003884(alphaTub84B)
		S.fur-22.63	/	FBgn0003886(alphaTub85E)
		S.fur-214.43,S.fur-9.290	NLU005541.1,NLU009958.1,NLU	FBgn0087040(alphaTub6

			020924.1,NLU008983.1	7C)
		/	/	FBgn0003885(alphaTub84D)
	cad	/	NLU005601.1	FBgn0000251
	Dlg5	S.fur-33.149	NLU006162.1,NLU022716.1	FBgn0032363
	dos	S.fur-19.265	NLU009379.1	FBgn0016794
	dup	/	NLU015058.1,NLU015059.1	FBgn0000996
	E2f1	/	NLU014009.1	FBgn0011766
	Eip75B	S.fur-40.72	NLU006196.1	FBgn0000568
	IMPPP	/	/	FBgn0033835
	kay	/	/	FBgn0001297
	lola	S.fur-124.39	NLU007358.1	FBgn0005630
	lysozyme	S.fur-11.139	NLU018928.1	FBgn0035813
		S.fur-145.15	NLU013788.1	/
		S.fur-53.22	NLU027010.1	/
		S.fur-85.10	NLU021394.1,NLU008673.1	/
		S.fur-85.9		/
		/	NLU017394.1	FBgn0025827,FBgn0034162,FBgn0034165,,FBgn0046999
		/	/	FBgn0034539,FBgn0029765,FBgn0034538

		/	/	FBgn0004425, FBgn0004426, FBgn0004427, FBgn0004428, FBgn0004429, FBgn0004430, FBgn0004431, FBgn0050062, FBgn0034092
		/	/	/
	mbo	S. fur-7. 118	/	FBgn0026207
	MED25	/	NLU002125. 3, NLU023106. 1	FBgn0038760
	Mef2	S. fur-35. 38	NLU018410. 1, NLU018409. 1	FBgn0011656
	Ntf-2	S. fur-9. 95	NLU013180. 3	FBgn0031145
	par-1	S. fur-14. 79	NLU011670. 2, NLU011671. 1	FBgn0260934
	Parp	S. fur-72. 107	NLU018225. 1, NLU027931. 1	FBgn0010247
	PCNA	S. fur-74. 129	NLU007941. 1	FBgn0005655
	smog	S. fur-56. 66	NLU022287. 1, NLU028367. 1	FBgn0051660
	Ptip	S. fur-49. 48	NLU019373. 1	FBgn0052133
	Pvr	S. fur-27. 55	NLU002246. 1, NLU004968. 1	FBgn0032006
		S. fur-170. 3		
	Rab11	S. fur-13. 193	NLU026714. 1	FBgn0015790
	scrib	S. fur-347. 18	NLU017397. 1	FBgn0263289
	Spt6	S. fur-98. 17	NLU022796. 3	FBgn0028982
	Tep1	S. fur-70. 51	NLU024076. 1	FBgn0041183
	Tep2			FBgn0041182

	Tep3			FBgn0041181
	Tep4			FBgn0041180
	Thor	S.fur-207.15	NLU001242.1,NLU015401.1	FBgn0261560
	Tlk	S.fur-682.3	/	FBgn0086899
	Vps15	S.fur-270.24	NLU026186.1,NLU002513.1	FBgn0260935
	vvl	S.fur-250.11	NLU025618.1	FBgn0086680
	zfh1	S.fur-36.98	NLU017934.2	FBgn0004606
antimicrobial peptide	Attacin-A	/	/	FBgn0012042
	Attacin-B	/	/	FBgn0041581
	Attacin-C	/	/	FBgn0041579
	Attacin-D	/	/	FBgn0038530
	Cecropin A1	/	/	FBgn0000276
	Cecropin A2	/	/	FBgn0000277
	Cecropin B	/	/	FBgn0000279
	Cecropin C	/	/	FBgn0000278
	Defensin	S.fur-10.120<br>S.fur-33.51	NLU011311.1,NLU007940.1	FBgn0010385
	Diptericin A	/	/	FBgn0004240
	Diptericin B	/	/	FBgn0034407
	Drosocin	/	/	FBgn0010388
	Drosomycin	/	/	FBgn0010381
	Drosomycin-like 1	/	/	FBgn0052274

	Drosomycin-like 2	/	/	FBgn0052279
	Drosomycin-like 3	/	/	FBgn0052283
	Drosomycin-like 4	/	/	FBgn0052282
	Drosomycin-like 5	/	/	FBgn0035434
	Drosomycin-like 6	/	/	FBgn0052268
	Metchnikowin	/	/	FBgn0014865
	NOS	S. fur-9.52	NLU025650.1	FBgn0011676
encapsulation of foreign target	alpha-Man-Ia	S. fur-17.159	NLU026219.1	FBgn0259170
	alpha-Man-Ib	S. fur-241.29	NLU014338.1	FBgn0039634
		S. fur-489.8		
	alpha-Man-IIa	S. fur-39.3	NLU004974.2	FBgn0011740
	alpha-Man-IIb	S. fur-129.28	NLU011543.1, NLU023574.1	FBgn0026616
		/		
		S. fur-825.7		
		S. fur-70.20		
	Als2	S. fur-494.2	NLU012777.1, NLU012778.1	FBgn0037116
	aPKC	S. fur-116.56	NLU007726.1	FBgn0261854
	Cdc42	S. fur-171.14	NLU025556.1	FBgn0010341
	Cdk5	S. fur-50.56	NLU014959.1	FBgn0013762
	Cdk5alpha	S. fur-150.22	NLU001312.1, NLU005199.1	FBgn0027491
		S. fur-150.19		
	DCTN1-p150	S. fur-85.11	NLU008672.1	FBgn0001108
	DCX-EMAP	S. fur-518.5	NLU003351.1	FBgn0259099
	Dhc64C	S. fur-63.19	NLU017272.1, NLU017748.1, NLU005440.1	FBgn0261797

	dia	S.fur-20.194	NLU006524.1	FBgn0011202
	Eb1	/	NLU007853.3	FBgn0027066
	Eph	S.fur-18.207	NLU018445.1,NLU018446.1	FBgn0025936
	Ephrin	S.fur-15.173	NLU007920.1	FBgn0040324
	Exn	S.fur-877.3	NLU011083.1	FBgn0261547
	Flo2	S.fur-229.21	NLU012648.1	FBgn0264078
	Itgbn	/	/	FBgn0010395
	LanA	S.fur-359.5	/	FBgn0002526
	Mgat1	/	NLU007274.1	FBgn0034521
	OstDelta	S.fur-223.18	NLU015744.1	FBgn0034277
	Ostgamma	S.fur-324.8	NLU017277.1	FBgn0032015
	poly	/	/	FBgn0086371
	RhoBTB	S.fur-33.154	NLU011755.1	FBgn0036980
	RhoGEF3	S.fur-18.90	NLU007117.1	FBgn0264707
	RhoL	/	/	FBgn0014380
	Shc	S.fur-11755.1	NLU026268.1	FBgn0015296
		S.fur-54.85		
		S.fur-54.99		
	Vav	S.fur-23.69	NLU028815.2	FBgn0040068
	Zir	S.fur-1433.1	NLU003059.2,NLU010547.1	FBgn0031216
		S.fur-440.5		
phagocytosis	crq	S.fur-51.4	NLU006788.1	FBgn0015924
		/	/	/
		S.fur-13.116	NLU028772.1	/
		/	/	FBgn0025697

	Rac1	/	/	FBgn0010333
	brm	S.fur-108.44<br>S.fur-598.8	NLU011826.1,NLU022334.1,NLU014082.1	FBgn0000212
	EcR	S.fur-50.12	NLU001978.1	FBgn0000546
	Jra	/	NLU005583.1,NLU018476.1	FBgn0001291
	pnt	S.fur-57.78	NLU015813.2	FBgn0003118
	betaCOP	S.fur-431.10	NLU010209.1	FBgn0008635
	Syx5	/	NLU017315.1	FBgn0011708
	gcm	S.fur-28.6<br>S.fur-84.70	/	FBgn0014179
	Trn	S.fur-69.5	NLU011103.1	FBgn0024921
	DMAP1	S.fur-85.55	NLU010679.1	FBgn0034537
	Nup98-96	S.fur-16.163<br>S.fur-1672.1<br>S.fur-1251.1	NLU007944.1	FBgn0039120
	psidin	/	NLU012476.2	FBgn0243511
	kuz	S.fur-192.21	NLU006855.1	FBgn0259984
mucosal immune response	bbg	S.fur-38.58	NLU017024.1	FBgn0087007
	Duox	S.fur-829.6<br>S.fur-921.1	NLU016480.1,NLU016482.1	FBgn0031464
	Galphaq	S.fur-72.236	NLU015017.2	FBgn0004435
	lic	S.fur-14.118	NLU003122.1	FBgn0261524
	Mekk1	S.fur-17.172	NLU002028.1	FBgn0024329
	Mkp3	S.fur-67.335	NLU004064.1	FBgn0036844
	norpA	S.fur-268.14	/	/

		S.fur-68.11	/	/
		S.fur-226.20	NLU020344.3	FBgn0004611
		/	/	FBgn0262738
		/	/	/
		/	/	/
		/	NLU005122.2,NLU019427.2	/
	p38a	S.fur-17.307	NLU014343.1	FBgn0015765
	p38b			FBgn0024846
negative regulation of immune response	Atg6	S.fur-596.2	NLU024452.1	FBgn0264325
	dnr1	S.fur-20.247	NLU011940.1	FBgn0260866
	Dsp1	S.fur-246.15	NLU015101.1,NLU021892.1	FBgn0011764
		S.fur-246.14		
	faf	S.fur-8.174	NLU006079.2,NLU012546.1,NLU021571.1	FBgn0005632
	GlyP	S.fur-487.5	NLU021959.1	FBgn0004507
	GstO2	/	NLU027429.1	FBgn0035906
	Lam	S.fur-197.34	NLU027896.1	FBgn0002525
	MED6	S.fur-295.17	NLU019881.1	FBgn0024330
		S.fur-951.1		
	Nxt1	/	/	FBgn0028411
	Prx5	S.fur-68.55	NLU020315.1	FBgn0038570
	Rala	S.fur-727.2	/	FBgn0015286
	RYBP	S.fur-7.142	NLU003809.1	FBgn0034763
	sax	S.fur-20.106	/	FBgn0003317

	scny	S.fur-84.32	NLU007457.1	FBgn0260936
	slif	S.fur-230.42	NLU019298.1,NLU020309.1	/
		S.fur-50.193	NLU023326.1	FBgn0036493,FBgn0036764
		S.fur-345.27	NLU000066.1,NLU020081.1	/
		/	/	FBgn0037203
		/	/	/
	SP2353	S.fur-197.44	/	FBgn0034070
	tefu	S.fur-158.19	NLU004916.1,NLU028531.1	FBgn0045035
	Tim10	S.fur-338.15	/	FBgn0027360
	unc-45	S.fur-97.14	NLU005097.1	FBgn0010812
	Usp2	S.fur-20.70	NLU013381.1	FBgn0031187
	Nrf2	S.fur-9.282	NLU002199.1	FBgn0262975
	wun	S.fur-273.10	NLU004856.2	FBgn0016078
positive regulation of immune response	akirin	/	/	FBgn0082598
	att-ORFA	S.fur-622.8	NLU019757.1	FBgn0067783
	CG13994	/	/	FBgn0031772
	CG17680	/	/	FBgn0062440
	CG2051	S.fur-16.19	NLU023404.1	FBgn0037376
	CG32795	S.fur-32.48	NLU021502.1	FBgn0040384
	CG9925	/	/	FBgn0038191
	chico	S.fur-77.62	NLU003485.1,NLU024952.1	FBgn0024248
	Crag	S.fur-89.92	NLU026389.1	FBgn0025864
	Etl1	S.fur-181.1	NLU020728.1	FBgn0032157
	HBS1	S.fur-134.8	NLU014461.1	FBgn0042712

	Hr78	S.fur-162.14	NLU015534.1	FBgn0015239
	htl	S.fur-27.19	/	FBgn0005592,FBgn0010389
	Ide	S.fur-36.56	NLU013696.3	FBgn0001247
	kug	S.fur-369.15	NLU005127.1,NLU018506.1,NLU018507.1	FBgn0001075
		S.fur-316.19	NLU008341.1	/
		S.fur-6.221	NLU010443.1,NLU016499.1	/
		S.fur-316.13	NLU015764.1,	FBgn0000497
		S.fur-6.194	NLU007013.1	/
		/	/	FBgn0261574
	LanB1	S.fur-24.172	NLU025937.2	FBgn0261800
	Mmp2	S.fur-259.18	NLU025213.1	FBgn0033438
	mtd	S.fur-8.143	NLU003015.1,NLU003011.1,NLU003010.1	FBgn0013576
	Nsun5	/	/	FBgn0259704
	PDCD-5	S.fur-169.12	NLU008889.1	FBgn0036580
	Pp1alpha-96A	/	NLU011463.1	FBgn0003134
	puc	S.fur-167.52	NLU023509.1,NLU016695.1	FBgn0243512
	RluA-2	/	NLU002857.1	FBgn0032256
	Sec31	S.fur-11.25	NLU003794.1,NLU003795.1	FBgn0033339
immune response	Atf3	S.fur-125.49	NLU020429.1	FBgn0028550
	cher	S.fur-7.140	NLU003806.1	FBgn0014141
	CSN5	S.fur-44.49	NLU027652.1	FBgn0027053
	Deaf1	S.fur-438.6	NLU009895.1	FBgn0013799

	Dlip3	/	/	FBgn0040465
	eya	S.fur-20.138	NLU019650.1	FBgn0000320
	modSP	S.fur-102.37	NLU026230.2	FBgn0051217
		S.fur-79.44		
	POSH	/	NLU011904.2,NLU024807.1	FBgn0040294
	Reeler	S.fur-159.27	NLU024648.1	FBgn0039648,FBgn0261534
	Uba2	S.fur-594.3	NLU006181.1	FBgn0029113
		S.fur-691.11		
	Vps16B	S.fur-97.22	NLU025446.3	FBgn0039702
	Vps33B	S.fur-107.10	/	FBgn0039335
Caspase	Caspase	S.fur-17.341	NLU005472.1	FBgn0028381
	Caspase	S.fur-6.178	NLU005810.1	FBgn0026404
	Caspase	S.fur-1194.2	NLU006137.1	FBgn0036967
	Caspase	S.fur-23.136	NLU002884.1	FBgn0010501
	Caspase	S.fur-739.1	/	FBgn0019972
	Caspase	S.fur-460.16	/	FBgn0036449
	Caspase	S.fur-24.353	/	FBgn0028406
	Caspase	/	/	FBgn0033051
	Caspase	/	/	FBgn0033659
	Caspase	/	/	FBgn0033661
	Caspase	/	/	FBgn0020381
Catalase	Catalase	/	/	FBgn0038465
	Catalase	S.fur-314.12	NLU001797.1	FBgn0000261
Fibrinogen-like	Fibrinogen-like	/	/	FBgn003016

Proteins	Proteins			FBgn0030164 FBgn0031804 FBgn0050281 FBgn0033312 FBgn0034160 FBgn0050280
	Fibrinogen-like Proteins	/		
	Fibrinogen-like Proteins	/	/	
	Fibrinogen-like Proteins	S.fur-418.10	NLU010459.1	
	Fibrinogen-like Proteins	S.fur-72.247	NLU023744.1	
	Fibrinogen-like Proteins	/	/	
	Fibrinogen-like Proteins	/	/	
	Fibrinogen-like Proteins	/	/	FBgn0087011
	Fibrinogen-like Proteins	/	/	FBgn0038365
	Fibrinogen-like Proteins	/	/	FBgn0003326
IAP Repeat	IAP Repeat	/	/	FBgn0037808
	IAP Repeat	S.fur-234.14	NLU004965.1	FBgn0260635
	IAP Repeat	S.fur-119.29	NLU001578.1	FBgn0015247
	IAP Repeat	/	/	FBgn0264291
MD-2-related lipid recognation	MD-2-related lipid recognation	/	/	FBgn0031381
	MD-3-related	/	/	FBgn0039801

	lipid recognation			
	MD-4-related lipid recognation	/	/	FBgn0051410
	MD-5-related lipid recognation	/	/	FBgn0037783
	MD-6-related lipid recognation	/	/	FBgn0037782
	MD-7-related lipid recognation	/	/	FBgn0038198
	MD-8-related lipid recognation	S. fur-283. 24	/	FBgn0039154
	MD-9-related lipid recognation	/	/	FBgn0039800
	MD-10-related lipid recognation	/	/	FBgn0039801
	MD-11-related lipid recognation	/	/	FBgn0039801
Scavenger Receptiors	Scavenger Receptiors	/	/	FBgn0015924
	Scavenger Receptiors	/	/	FBgn0025697
	Scavenger Receptiors	S. fur-28. 74	NLU001450. 2	FBgn0010435
	Scavenger Receptiors	S. fur-65. 66	NLU016424. 2	FBgn0014033
	Scavenger	S. fur-101. 93	NLU015195. 1	FBgn0020377

	Receptiors			
	Scavenger Receptiors	S.fur-84.65	NLU029081.1	FBgn0031547
	Scavenger Receptiors	S.fur-38.96	NLU017261.1	FBgn0020376
	Scavenger Receptiors	S.fur-982.3	NLU011924.1	FBgn0034660
	Scavenger Receptiors	S.fur-113.6	NLU028762.1	FBgn0027562
	Scavenger Receptiors	S.fur-74.3	NLU005452.1	FBgn0023479
	Scavenger Receptiors	S.fur-168.24	NLU009452.1	FBgn0031571
	Scavenger Receptiors	S.fur-17.355	NLU020729.3	FBgn0260004
	Scavenger Receptiors	S.fur-15.137	NLU009453.1	FBgn0033192
	Scavenger Receptiors	S.fur-1.36	NLU016193.1	FBgn0035091
	Scavenger Receptiors	S.fur-15.133	NLU011927.1	FBgn0058006
	Scavenger Receptiors	/	/	FBgn0035815
	Scavenger Receptiors	/	/	FBgn0035090
Spatzle	Spatzle	S.fur-54.115	NLU023813.1	FBgn0003495

	Spatzle	S.fur-5.212	NLU021599.1	FBgn0035056
	Spatzle	S.fur-24.293	NLU021391.1	FBgn0031959
	Spatzle	S.fur-166.29	NLU021217.1	FBgn0035379
	Spatzle	S.fur-261.1	NLU009098.1	FBgn0052343
	Spatzle	S.fur-72.508	NLU004373.1	FBgn0032362
	Spatzle	S.fur-33.150	NLU006163.1	FBgn0085438
	Spatzle	/	NLU019692.1	FBgn0039102
	Spatzle	/	NLU021596.1	/
Superoxide dismutase	Superoxide dismutase	S.fur-13.25	/	/
	Superoxide dismutase	S.fur-41.88	/	FBgn0034962
	Superoxide dismutase	S.fur-8.79	/	FBgn0039386
	Superoxide dismutase	S.fur-23.71	NLU015321.2	FBgn0003462
	Superoxide dismutase	S.fur-33.90	NLU000900.1	FBgn0010213
	Superoxide dismutase	S.fur-67.126	NLU014615.1	FBgn0033631
	Superoxide dismutase	S.fur-67.143	NLU011195.1	FBgn0052850
Thioester-containing protein	Thioester-containing protein	S.fur-70.51	NLU024076.1	FBgn0041180
	Thioester-containing protein	/	NLU015403.1	FBgn0041182

	Thioester-containing protein	/	/	FBgn0041181
	Thioester-containing protein	/	/	FBgn0041183
	Thioester-containing protein	/	/	FBgn0039854
	Thioester-containing protein	/	/	FBgn0039855
peroxidase	peroxidase	S.fur-219.18	NLU019777.1	FBgn0038511
	peroxidase	S.fur-126.46	NLU001846.1	FBgn0010053
	peroxidase	S.fur-46.51	NLU003657.1	FBgn0035438
	peroxidase	S.fur-77.96	NLU020527.1	FBgn0031464
	peroxidase	S.fur-18.30	NLU024356.2	FBgn0032685
	peroxidase	S.fur-120.36	NLU021034.1	FBgn0040309
	peroxidase	S.fur-273.7	NLU011776.1	FBgn0038519
	peroxidase	S.fur-47.125	NLU015971.1	FBgn0040305
	peroxidase	S.fur-30.107	NLU026271.1	FBgn0037102
	peroxidase	S.fur-15.277	NLU025371.1	FBgn0040308
	peroxidase	S.fur-65.41	NLU026550.1	FBgn0259233
	peroxidase	S.fur-17.242	NLU003276.1	FBgn0033520
	peroxidase	S.fur-430.8	NLU021340.1	FBgn0033521
	peroxidase	S.fur-6.74	NLU004434.1	FBgn0033518
	peroxidase	S.fur-22.68	NLU016990.1	FBgn0031479
	peroxidase	S.fur-128.19	NLU006818.1	FBgn0038465
	peroxidase	S.fur-22.79	NLU019027.2	FBgn0011828

Table S3. Summary of the 11 RNA-seq libraries used in this study

Sample		Clean Reads	Clean Base (G)	Total Genome Map reads	Total Genome Map Rate (%)	Uniq Genome Map Reads	Uniq Genome Map Rate (%)	SRBSDV Map Rate (%)	Total mapping（%）
HVT	C1	22,037,322	6.04	4,828,667	21.91%	3,050,280	13.84%	38.31%	60.22%
	C2	24,570,532	6.68	5,831,096	23.73%	3,226,271	13.13%	26.70%	50.43%
MVT	A1	32,580,835	8.96	7,366,039	22.61%	4,124,258	12.66%	25.35%	47.96%
	A2	27,972,323	7.70	6,443,219	23.03%	3,327,296	11.89%	23.30%	46.33%
	A3	28,857,802	7.91	7,651,278	26.51%	4,250,958	14.73%	15.53%	42.04%
NVF	B1	32,940,740	8.92	20,507,542	62.26%	10,686,618	32.44%	0.12%	62.38%
	B2	29,828,260	8.00	17,682,796	59.28%	9,013,809	30.22%	0.13%	59.41%
	B3	33,855,507	9.25	15,111,096	44.63%	9,008,779	26.61%	0.16%	44.79%
Healthy	O1	19,263,210	5.30	11,875,751	61.65%	8,717,036	45.25%	0.09%	61.74%
	O2	19,934,219	5.49	12,153,259	60.97%	8,134,962	40.81%	0.09%	61.06%
	O3	20,968,250	5.76	12,795,336	61.02%	9,040,440	43.11%	0.07%	61.09%

Table S4. The down- regulated and up-regulated immune-related genes in NVF, MVT and HVT

Down/Up-regulated	Gene ID	Gene name	NFV	MVT	HVT
Down-regulated	Sfur-10.120	Defensin	Yes	No	Yes
	Sfur-107.10	immune response	Yes	Yes	Yes
	Sfur-166.29	Spatzle	No	Yes	Yes
	Sfur-18.207	encapsulation of foreign target	No	Yes	No
	Sfur-184.28	IMD signaling pathway	No	Yes	No
	Sfur-197.44	negative regulation of immune response	No	Yes	Yes
	Sfur-219.18	Peroxidase	Yes	Yes	Yes
	Sfur-22.68	Peroxidase	Yes	Yes	Yes
	Sfur-22.79	Peroxidase	Yes	Yes	Yes
	Sfur-295.17	negative regulation of immune response	Yes	No	No
	Sfur-430.8	Peroxidase	No	Yes	No
	Sfur-49.2	CLIP-domain serine protease	No	Yes	No
	Sfur-54.75	CLIP-domain serine protease	Yes	No	No
	Sfur-602.5	Atg7	No	No	Yes
	Sfur-65.41	Peroxidase	No	Yes	No
	Sfur-67.126	Superoxide dismutase	Yes	Yes	Yes
	Sfur-74.3	Scavenger Receptiors	Yes	Yes	Yes
	Sfur-829.6	mucosal immune response	Yes	Yes	Yes
	Sfur-925.2	C-lectin	No	Yes	No
Up-regulated	Sfur-11755.1	encapsulation of foreign target	No	Yes	Yes

	Sfur-126.46	Peroxidase	Yes	No	No
	Sfur-24.293	Spatzle	Yes	No	No
	Sfur-2570.1	Serpin	Yes	Yes	Yes
	Sfur-335.4	Serpin	No	Yes	No
	Sfur-377.2	Serpin	No	No	Yes
	Sfur-418.10	Fibrinogen-like Proteins	Yes	No	No
	Sfur-54.85	encapsulation of foreign target	Yes	Yes	Yes
